# Admixture as a source for HLA variation in Neolithic European farming communities

**DOI:** 10.1186/s13059-025-03509-6

**Published:** 2025-02-28

**Authors:** Nicolas Antonio da Silva, Onur Özer, Magdalena Haller-Caskie, Yan-Rong Chen, Daniel Kolbe, Sabine Schade-Lindig, Joachim Wahl, Carola Berszin, Michael Francken, Irina Görner, Kerstin Schierhold, Joachim Pechtl, Gisela Grupe, Christoph Rinne, Johannes Müller, Tobias L. Lenz, Almut Nebel, Ben Krause-Kyora

**Affiliations:** 1 Institute of Clinical Molecular Biology, Kiel University, Kiel, Germany; 2 Research Unit for Evolutionary Immunogenomics, Department of Biology, University of Hamburg, Hamburg, Germany; 3 Landesamt für Denkmalpflege Hessen, hessenARCHÄOLOGIE, Schloss Biebrich, Wiesbaden, Germany; 4 Institute for Archaeological Sciences, Palaeoanthropology Working Group, University of Tübingen, Tübingen, Germany; 5 Anthropologische Dienstleistungen Konstanz, Constance, Germany; 6 Landesamt für Denkmalpflege im Regierungspräsidium Stuttgart, Constance, Germany; 7 Museumslandschaft Hessen Kassel, Sammlung Vor- und Frühgeschichte, Kassel, Germany; 8 LWL-Altertumskommission für Westfalen, Münster, Germany; 9 Institut für Archäologien, Universität Innsbruck, Innsbruck, Austria; 10 Biocenter of the Ludwig Maximilian University, Munich, Germany; 11 Institute of Pre- and Protohistoric Archaeology, Kiel University, Kiel, Germany

**Keywords:** Ancient DNA, European Neolithic, Population genetics, Admixture, Immune genes, HLA diversity

## Abstract

**Background:**

The northern European Neolithic is characterized by two major demographic events: immigration of early farmers from Anatolia at 7500 years before present, and their admixture with local western hunter-gatherers forming late farmers, from around 6200 years before present. The influence of this admixture event on variation in the immune-relevant human leukocyte antigen (HLA) region is understudied.

**Results:**

We analyzed genome-wide data of 125 individuals from seven archeological early farmer and late farmer sites located in present-day Germany. The late farmer group studied here is associated with the Wartberg culture, from around 5500–4800 years before present. We note that late farmers resulted from sex-biased admixture from male western hunter-gatherers. In addition, we observe Y-chromosome haplogroup I as the dominant lineage in late farmers, with site-specific sub-lineages. We analyze true HLA genotypes from 135 Neolithic individuals, the majority of which were produced in this study. We observe significant shifts in HLA allele frequencies from early farmers to late farmers, likely due to admixture with western hunter-gatherers. Especially for the haplotype DQB1*04:01-DRB1*08:01, there is evidence for a western hunter-gatherer origin. The HLA diversity increased from early farmers to late farmers. However, it is considerably lower than in modern populations.

**Conclusions:**

Both early farmers and late farmers exhibit a relatively narrow HLA allele spectrum compared to today. This coincides with sparse traces of pathogen DNA, potentially indicating a lower pathogen pressure at the time.

**Supplementary Information:**

The online version contains supplementary material available at 10.1186/s13059-025-03509-6.

## Background

Since the Paleolithic, central Europe had been populated by western hunter-gatherers (WHG). Around 7500 before present (BP), the first farmers arrived who originated from Anatolia, bringing with them agriculture as subsistence and the Neolithic lifestyle [1, 2]. Archeologically, these early European farmers (EF) are associated with Linear Pottery societies (Linearbandkeramik, LBK, ~ 7500–6900 BP). LBK and subsequent societies remained largely unadmixed with WHG, as reflected in their high genetic similarity to the Anatolian source population [3, 4]. The rate of admixture gradually increased from the Younger and Late Neolithic (6200–4800 BP) onwards, so that the gene pool of the resulting late farmers (LF) contained a substantial WHG ancestry component [3–6]. These demographic and genomic changes coincided with cultural transformations that led to the dissolution of LBK/post-LBK societies and ultimately to the emergence of many small and regionally diverse societies, such as the one affiliated with the Wartberg context (WBC, ~ 5500–4800 BP) [6–8]. So far, only one WBC burial community (i.e., Niedertiefenbach in Germany, 5300–5200 BP) has been comprehensively studied by ancient genomics [6]. This group had a surprisingly high WHG ancestry (34–58%) and a distinct human leukocyte antigen (HLA) allele profile that was mainly focused on the detection of viral infections [6]. However, whether these genomic characteristics of the Niedertiefenbach population were typical of the WBC in general remains to be clarified. Another question is to what extent the HLA repertoire of the WBC-associated farmers differed from that of earlier groups, for instance, LBK and post-LBK communities.

HLA molecules play a key role in adaptive immunity and exhibit exceptional levels of polymorphism, presumably driven by pathogen-mediated selection [9–11]. Recent studies have used dense SNP data to uncover the evolutionary history of the HLA region [12–14]. However, this approach is limited to HLA alleles that can be tagged by SNPs that have been identified in modern populations (i.e., relying on the assumption that linkage disequilibrium has not changed since prehistoric times). By capturing the HLA genes and developing genotyping pipelines suitable for low-coverage data, it has been possible to generate true HLA calls, providing initial glimpses into the immunogenetic makeup of Neolithic populations [6, 15].

**Fig. 1:**
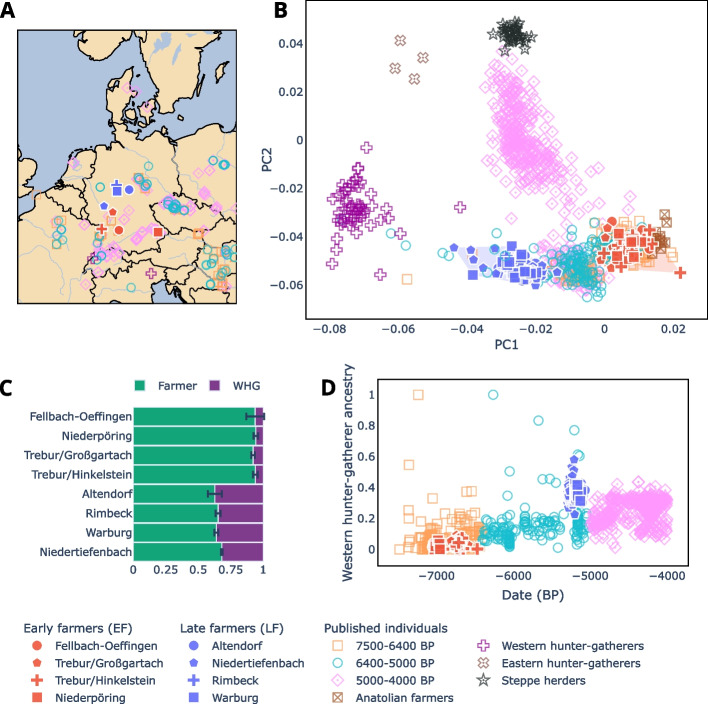
Genetic, temporal and geographic information.** A** Map showing locations of the Early (red) and Late (blue) Neolithic sites included in this study. **B** Principal component analysis of ancient individuals projected onto modern West-Eurasian variation (modern samples not shown). Convex hulls highlight the space filled by early farmers (EF) and late farmers (LF). **C** Ancestry proportions based on the average of feasible qpAdm models using multiple western hunter-gatherer (WHG) and farmer reference populations. Error bars represent standard errors across the selected models. **D** Timeline of studied samples and their WHG ancestry estimated with supervised ADMIXTURE

Here, we performed population genetic analyses using newly generated genome-wide data of 83 individuals from six archeological sites in Germany covering the 2300-year time span from the Early (LBK) to the Late Neolithic (WBC) (Fig. 1A, Table 1). We also included previously published data from the WBC population of Niedertiefenbach (*n* = 42) [6]. Moreover, we analyzed HLA genotype calls of 135 Neolithic individuals produced by us from all seven sites (Table 1), thus significantly expanding the publicly available data [6, 15, 16]. The large sample size provided more reliable HLA allele frequencies and allowed us to perform robust and informative comparisons between Neolithic and modern populations.

**Table 1:** Archeological context of the sites and populations used in this study

Site	Dating (calBP)	Culture	Sample size # skeletal remains	Sample size for population genetics	Sample size for HLA analysis
Fellbach-Oeffingen	7000 [17, 18]	Linear Pottery	34	14	17
Niederpöring	7000 [19]	Linear Pottery	14	6	6
Trebur [20–25]	7000–6500 [26, 27]	Hinkelstein	50	17	13
Trebur [20–25]	7000–6500 [26, 27]	Großgartach	28	12	9
Altendorf [28–31]	5250–5100 [32]	Wartberg	21	15	13
Warburg [33–39]	5300–4900 [40]	Wartberg	18	17	18
Rimbeck [41–43]	5200^a^ [33]	Wartberg	10	2	3
**Subtotal**			**175**	**83**	**79**
Niedertiefenbach (this study and [6])	5300–5200 [6]	Wartberg	89	42	56^b^
**Total**			**264**	**125**	**135**

^a^Data provided as personal communication from C. Rinne

^b^This number includes previously published HLA profiles (*n* = 23; Immel et al. 2021 [6]) plus calls newly generated in this study (*n* = 33)

## Results

### Studied individuals are grouped by genetic affinity

In this study, we generated shotgun sequences from the remains of 175 individuals originating from the following six sites in present-day Germany: Fellbach-Oeffingen, Niederpöring, Trebur, Altendorf, Warburg and Rimbeck (Table 1; Fig. 1A; Additional file 1: Data S1; archeological context information available in Additional file 2: Note S1). The Trebur site contained burials assigned to the Middle Neolithic Hinkelstein and Großgartach groups [26, 44, 45].

When mapping the shotgun sequencing data to the human genome build hg19 (summary statistics available in Additional file 1: Data S2), 83 of the 175 samples had at least 20,000 SNPs of the 1240 K panel covered and were considered for the subsequent population genetic analyses (Table 1; Additional file 1: Data S1). In addition, we included previously published data from the Niedertiefenbach collective (*n* = 42) [6], resulting in a total of 125 genome-wide datasets for population genetic analyses. We first conducted a principal component analysis (PCA), projecting ancient samples onto the variation observed in modern West-Eurasian populations. This analysis revealed that the seven populations formed two distinct groups: individuals from Fellbach-Oeffingen, Niederpöring, and Trebur clustered with published Early Neolithic farmers, whereas individuals from Altendorf, Warburg, Rimbeck, and Niedertiefenbach (representing WBC) were placed near agriculturalists from the Late Neolithic (Fig. 1B; Additional file 2: Fig. S1). Correspondingly, outgroup f3 statistics showed that our Early Neolithic individuals had higher genetic affinities with contemporaneous groups from the LBK, Sopot, and Starčevo societies (Additional file 2: Fig. S2), while the WBC groups were more similar to WHG proxies (i.e., Loschbour Luxembourg, Bichon Switzerland, and one individual from Mont Aimé/Paris Basin). Therefore, we refer to the former group (Fellbach-Oeffingen, Niederpöring and Trebur; *n* = 49) as early farmers (EF) and the latter (Altendorf, Warburg, Rimbeck and Niedertiefenbach; *n* = 76) as late farmers (LF). This classification is consistent with their archeological dates (Table 1).

### Late farmers of the WBC group showed unusually high WHG ancestry

Next, we conducted several admixture analyses to gain deeper insights into the genetic structure of the seven populations. Unsupervised ADMIXTURE analysis revealed that both EF and LF carried two major ancestry components, one maximized in WHG and the other in Anatolian Neolithic farmers (AN) (Additional file 2: Fig. S3-4). This was supported by qpWave modelling using two sources: Luxembourg_Loschbour (WHG proxy) and Turkey_N (AN) (rank1 *p*-value > 0.05; Additional file 1: Data S3). We then tested two-way qpAdm models to obtain ancestry proportions with several WHG and AN proxies as sources. Averaging the estimates from feasible models (i.e., with *p* ≥ 0.05 and where the proportions were in the interval [0, 1]), we observed a much lower WHG component in EF (6%) than in LF (35%) (Fig. 1C; Additional file 1: Data S4). We additionally tested three-way qpAdm models by incorporating a steppe proxy (Russia Samara EBA Yamnaya) as a third source to examine potential gene flow from the steppe into our LF groups, whose dating begins to overlap with the Yamnaya horizon. The results indicated virtually no admixture with populations carrying the steppe-related ancestry component (Additional file 1: Data S4). Admixture date modelling with DATES using WHG and AN as sources revealed that the most recent WHG introgression into LF probably occurred between 6500 and 5500 BP (Additional file 1: Data S5; Additional file 2: Fig. S5). Supervised ADMIXTURE analysis showed that LF, representing WBC in this study, exhibited unusually high WHG ancestry compared to contemporaneous groups (Fig. 1D). Other individuals with a WHG ancestry as high as in WBC were only observed in Blätterhöhle (I1563, I1565, I1593, I1594) and as single outliers in various Late Neolithic groups (e.g., Mont Aimé (2H11), Makotřasy (I7193), Tangermünde (TGM009); samples highlighted in Additional file 2: Fig. S1). For the two Trebur subgroups, the unsupervised admixture and the PCA analyses suggested a slight difference in the amount of WHG ancestry. However, individual qpADM modelling did not support this (Additional file 1: Data S6; Additional file 2: Fig. S6).

### Intra-group kinship was limited

We explored the possibility of kinship within each group by calculating the relatedness coefficient based on pairwise mismatch rates. Mitochondrial (mt) DNA and Y-chromosome haplogroups were also considered in the analysis (Additional file 1: Data S1). We identified a few cases of 1st- or 2nd-degree kinship in Altendorf (*n* = 2 relationships), Fellbach-Oeffingen (*n* = 1), and Trebur/Hinkelstein (*n* = 6) (Additional file 2: Fig. S7 and Table S1). For Niedertiefenbach, we have previously reported three children who were siblings [6]. Consequently, from each pair/triplet of 1st degree relatives, the individual with the more complete HLA profile was used for the HLA frequency calculations. Strikingly, we noted large sex differences in the four WBC communities, with females (*n* = 24 out of 81) representing only 30% of the buried population.

### Haploid lineages changed from EF to LF

We observed changes in the distribution of both mtDNA and Y-chromosome haplogroups between EF and LF (Additional file 2: Fig. S8). For mtDNA, the shifts (increase in U5 and H; decrease in K and T) are consistent with previous findings [3, 6, 46, 47]. Interestingly, LF had only one Y-chromosome macro-lineage (I), whereas EF had five. Macro-lineage I is common in WHG [46, 48]. Higher-resolution I haplotypes were available for 16 LF men in our data (Additional file 1: Data S1), which were further subdivided into only three sub-lineages: I2a1a (*n* = 5), I2a2 (*n* = 1), and I2c (*n* = 10). I2c was the dominant lineage in Niedertiefenbach and Altendorf and I2a1a in Warburg.

### WHG male-biased admixture formed WBC-associated late farmers

The imbalance in the diversity of mtDNA and Y-chromosome haplogroups in the WBC-associated LF could indicate a sex bias during the admixture event that formed LF. Therefore, we tested whether this was the case. First, we explored the statistic Q which measures relative genetic drift between the X-chromosome and autosomes. Q is expected to be 0.75 if the effective population size of males and females is equal. Deviations from this value may be suggestive of a sex-biased demography. Comparing AN and LF yielded results compatible with the expected value (Q = 0.76), while the WHG–LF comparison suggested a slight deviation (Q = 0.63) (Additional file 2: Table S2). We then computed the ancestry proportions on the X-chromosome and autosomes separately and calculated the ratio of X-chromosome to autosome WHG ancestry (which we here refer to as R_X/A_). An equal admixture contribution of males and females should lead to R_X/A_ = 1, while deviations from this may be indicative of male- or female-biased admixture. We observed R_X/A_ < 1 in 24 out of 38 individuals (63%) that entered the analysis (mean R_X/A_ = 0.8; median R_X/A_ = 0.87; right-sided binomial test, *p* = 0.0717; Additional file 1: Data S7; Additional file 2: Fig. S9). Interestingly, a few individuals (*n* = 14; 37%) showed drastic deviations (R_X/A_ < 0.5) from the expected value of 1. The distributions of X-chromosome and autosome WHG ancestry in LF were significantly different (median *p* = 0.01, Wilcoxon signed-rank test; Additional file 2: Fig. S10), suggestive of WHG male-biased admixture (i.e., more male WHG ancestors).

### Late farmers exhibited low effective population size

We investigated the amount of runs of homozygosity (ROH) between EF and WBC-associated LF. It was possible to infer ROH for 6 EF and 39 LF individuals (Additional file 1: Data S8; Additional file 2: Fig. S11). EF individuals presented on average shorter ROH (6 cM) than LF (12 cM). When we included data from published LBK sites (*n* = 24 individuals; results generated in [49]) in the EF group to increase the sample size, the average ROH remained in the same range (5 cM). We observed a statistically significant difference in the sum ROH (in the range 4–8 cM) between EF (including published LBK data) versus LF (*p* = 0.04884). ROH ≥ 20 cM were found in one individual associated with LBK and two LF individuals. We also used ROH to estimate the effective population sizes (N_e_), which showed significantly higher values for EF (including published LBK data) (N_e_ = 7570, 95% CI = 5105–11,950) than for LF (N_e_ = 3371, 95% CI = 2665–4384).

### Early and late farmers differed in their HLA allele pools

**Fig. 2:**
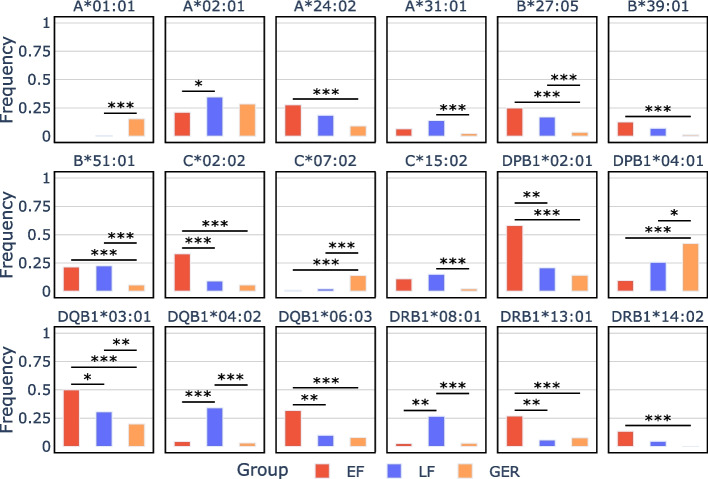
HLA alleles showing significant frequency differences between early farmers (EF) and late farmers (LF) or between either Neolithic group and modern Germans (GER). The Y-axis represents allele frequencies calculated separately for each HLA locus (i.e., HLA-A, -B, -C, -DPB1, -DQB1, and -DRB1). Only alleles with statistically significant changes (*p* ≤ 0.05 and absolute frequency difference ≥ 0.1) are displayed. Significance was assessed using Fisher’s exact test with multiple test correction (*: *p* ≤ 0.05; **: *p* ≤ 0.01; ***: *p* ≤ 0.001)

For the seven populations, we enriched and sequenced the three HLA class I (HLA-A, -B, -C) and three class II loci (HLA-DPB1, -DQB1, -DRB1). The data were merged with those from the shotgun sequencing, resulting in sufficient HLA coverage to genotype 112 unrelated individuals (Table 1). Through inclusion of previously published genotypes from 23 Niedertiefenbach individuals [6], we achieved a total of 135 HLA profiles (EF = 45, LF = 90) with varying levels of coverage per locus (Additional file 1: Data S9; Additional file 2: Fig. S12 and Table S3). The data was used for allele frequency calculations (genotypes available in Additional file 1: Data S1; allele frequencies in Additional file 1: Data S10 and Additional file 2: Fig. S13). We observed significant changes in eight HLA alleles between EF and LF (*p* ≤ 0.05, Fisher’s exact test corrected for multiple testing, Fig. 2, Additional file 2: Table S4). We additionally found major changes (≥ 10% frequency difference) in 17 alleles between the Neolithic groups (either EF or LF) and a representative sample of modern Germans [50] (*p* ≤ 0.05, Fisher’s exact test corrected for multiple testing; Fig. 2; Additional file 2: Table S4). These findings remained significant even after applying a down-sampling approach to control for differences in population sizes (Additional file 2: Fig. S14). The largest frequency changes (≥ 20%) in both comparisons affected mostly the HLA class II loci. Furthermore, we noted the co-occurrence of some HLA class II alleles, indicating haplotypes such as DRB1*13:01-DQB1*06:03 with 19% frequency in EF and DRB1*08:01-DQB1*04:02 with 20% frequency in LF. Out of the 20 most common HLA alleles in modern Germans (≥ 10%), seven alleles were not observed in our Neolithic samples and six were present only at low frequencies (< 5%) (Additional file 2: Table S5).

### HLA allele pool of late farmers was influenced by WHG ancestry

**Fig. 3:**
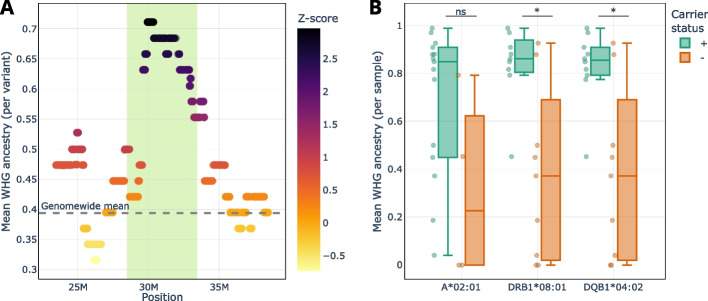
Enrichment of western hunter-gatherer (WHG) ancestry in the MHC region of late farmers (LF).** A** Local estimates of WHG ancestry in the MHC region (highlighted in green; chr6:28,477,797–33,448,354) of LF, along with the 5-Mb flanking regions on either side. The *Y*-axis shows the average probability of WHG ancestry for each variant in high-coverage LF individuals (*n* = 19) used in the local ancestry analysis. The grey dashed line represents the mean genome-wide WHG ancestry.** B** Comparison of the mean WHG ancestry across the MHC region between carriers (green) and non-carriers (orange) of alleles A*02:01, DRB1*08:01, and DQB1*04:02. The *Y*-axis represents the mean WHG ancestry probability of variants in the MHC region per individual. Significance of the difference in mean WHG ancestry between carriers and non-carriers of each allele was assessed using the Mann–Whitney test, with multiple testing correction applied (*: *p* ≤ 0.05; ns: not significant)

To assess whether the increased WHG ancestry in our LF population affected their HLA allele pool, we conducted a local ancestry analysis using a set of high-coverage imputed samples (LF = 19 individuals; WHG = 10, AN = 10; samples listed in Additional file 1: Data S11). We observed a substantial excess of WHG ancestry in LF within the larger major histocompatibility complex (MHC) (Fig. 3A). MHC consists of a 5-Mb region on chromosome 6 encompassing HLA and other immune-related genes. Subsequently, we tested whether the alleles that were more frequent in LF than EF (A*02:01, DRB1*08:01, DQB1*04:02; Fig. 2) were enriched for WHG ancestry. Our analysis revealed that this was indeed the case for individuals carrying DRB1*08:01 and DQB1*04:02 (Fig. 3B).

### Neolithic farmers showed low HLA diversity compared to modern Europeans

**Fig. 4:**
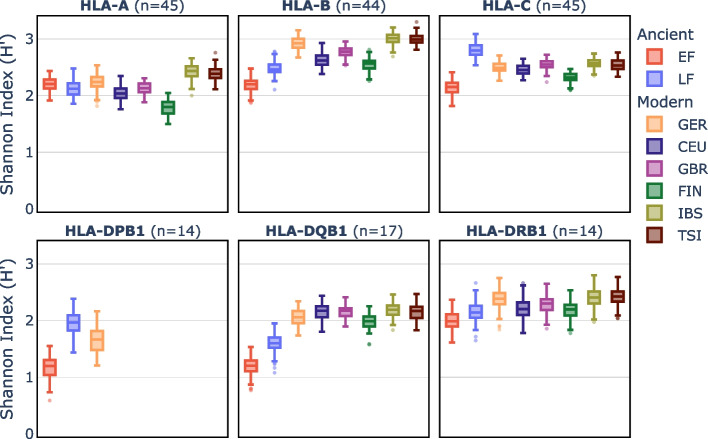
Comparison of diversity in the several HLA loci between Neolithic and modern populations. The *Y*-axis represents the HLA diversity as measured by the Shannon index (H’) for the loci A, B, C, DPB1, DQB1, and DRB1. Boxplots represent the distribution of the H’ values of 100 samples taken from each population with sample sizes n indicated in parenthesis beside the locus name. EF = early farmers; LF = late farmers; GER = modern Germans; CEU = central Europeans; GBR = British; FIN = Finnish; IBS = Iberians from Spain; TSI = Tuscans from Italy

We applied the Shannon diversity [51] index to quantify the HLA allele diversity within each population. Subsequently, we compared the HLA diversity between EF and LF and between either Neolithic group or modern Europeans (Germans [50] and five populations with European ancestry from the 1000 Genomes dataset [52]). The diversity was consistently and significantly lower in EF compared to LF for all loci with the exception of HLA-A (Fig. 4, Additional file 1: Data S12). Similarly, both EF and LF had lower diversities relative to modern populations except for HLA-A, HLA-C, and HLA-DPB1. The most drastic difference was observed in HLA-DQB1 that showed a remarkably low diversity in both Neolithic groups, owing to the dominance of a few HLA-DQB1 alleles. The two most common HLA-DQB1 alleles in EF and LF reached cumulative frequencies of 80% and 65%, respectively, while a more even distribution of frequencies was observed in modern Germans (Additional file 2: Fig. S13E).

### Few viral pathogens were found in Neolithic farmers

The shotgun sequencing data were screened for the presence of human blood-borne bacterial and viral pathogens. In two individuals from Niederpöring, reads of hepatitis B virus (NP560) and parvovirus B19 (NP543) were detected (Additional file 1: Data S1). No evidence of pathogens was found in any of the other samples.

## Discussion

Here, we analyzed genome-wide data from 49 early farmers (EF) and 76 late farmers (LF, from the WBC context) (Table 1, Fig. 1A). Our analyses showed that EF, like individuals from other published LBK sites in Germany [4, 53, 54], closely resembled Anatolian farmers (70–100% Anatolian ancestry component). In addition, they carried mtDNA and Y-chromosome lineages characteristic of early farming populations [3, 46, 48] (Additional file 2: Fig. S9). The genetic continuity throughout the LBK indicates long-lasting intracultural mating practices. However, close-kin mating was likely prevented as the analysis of ROH and N_e_ suggested a large group size and a wide partner exchange network as reported elsewhere [15, 49].

Previously, we have identified a large WHG ancestry component in individuals from the Niedertiefenbach community [6]. In the current study, which is based on a greater number of WBC individuals and sites, we confirmed that this feature is characteristic of all WBC-associated LF studied here (averaging 35% across all four sites; Fig. 1C). This proportion is unusually high compared with contemporaneous groups in the region, only matched by Blätterhöhle individuals as well as a few outliers from Late Neolithic groups (Fig. 1A, D; Additional file 2: Fig. S1). Furthermore, our analyses in LF individuals suggested a statistically significant male bias from WHG during their admixture with EF (Additional file 2: Fig. S8-10). LF presented more and longer ROH than EF (Additional file 2: Fig. S11). One explanation for this finding could be recent admixture with WHG introducing longer ROH. Another scenario could be the mating of relatives. However, the latter is not supported by our data, as mostly unrelated individuals were detected in the four collective burials studied here (Altendorf, Warburg, Rimbeck and Niedertiefenbach). We hypothesize that during the EF to LF transition, farming communities appear to have changed from closed to more permeable societies that were willing and able to integrate WHG, a process that was further accompanied by the diversification and regionalization of archeologically defined groups.

The introgression of WHG ancestry may also have led to changes in the mtDNA and Y-chromosome lineages (Additional file 2: Fig. S8). Of particular interest in this context are the Y-chromosomal shifts; in the WBC-associated LF individuals (Additional file 1: Data S1), we could only detect macro-lineage I. In addition, there appear to be community-specific patrilineages, as already observed for Niedertiefenbach (I2c) [6]. I2c was also the major lineage in Altendorf, whereas I2a1a dominated in Warburg. The presence of patrilineages is noteworthy in a population of unrelated individuals. This pattern could be suggestive of limited mating opportunities for males not carrying the patrilineage characteristic of a community. Overall, the WBC group appears to have been largely founded by men with I lineages.

Our genetic dating of the most recent admixture event between WHG and EF (Additional file 2: Fig. S5) confirmed our previous results that WBC (LF) most likely emerged from a Michelsberg context (MC; 6400–5500 BP) [6]. Although the estimated admixture date is based on a model that assumes a single pulse of admixture, a more complex scenario where WHG introgression occurred continuously or as multiple events is likely. There is evidence suggesting that the MC farmers were particularly mobile. For example, some MC groups used flint from non-local quarries, indicating that they were engaged in long-distance barter. In addition, they practiced forest pasture management which can be interpreted as transhumance [55–57]. This mobility may have led MC people to increasingly engage with WHG, contributing to the admixture of both groups. It is possible that the cultural characteristics of the admixed WBC groups were influenced in part by the relative contributions of each ancestral population (i.e., WHG and LBK). In any case, the admixture represented a profound transformation with long-lasting effects on demography, gene pool, and culture in Europe.

Next, we investigated whether EF and LF differed in their HLA variation. We observed significant frequency differences in eight HLA alleles, with the most pronounced changes occurring in class II (Fig. 2; Additional file 2: Table S4). As EF and LF varied in their proportion of WHG ancestry, the most plausible explanation for the considerable frequency shifts is admixture with WHG. A recent SNP-based study [12] has revealed that especially DQB1 is enriched for WHG ancestry in Late Neolithic individuals. This is supported by our results showing that LF had a substantial excess of WHG ancestry in the MHC region compared to genome-wide levels (Fig. 3A). Furthermore, our local ancestry analysis combined with HLA genotypes allowed us to identify an introgressed haplotype formed by DRB1*08:01 and DQB1*04:02 (Fig. 3B). The WHG gene flow also led to an increase in HLA diversity from EF to LF for most loci (Fig. 4). Admixture-enabled selection has been shown to drive specific HLA alleles towards higher frequencies [58, 59]. This process was also likely responsible for the increase in frequency of specific HLA class II alleles (such as DRB1*08:01 and DQB1*04:02) observed in LF. While the DRB1*08:01-DQB1*04:02 haplotype increased from EF to LF, another haplotype, DRB1*13:01-DQB1*06:03, decreased (Additional file 2: Fig. S13). Both were shown to be protective against hepatitis B virus (HBV) infections today [60–62]. It appears that the increase/decrease in the two haplotypes is proportional, indicating that the functional protective effect against HBV was maintained. Ancient DNA (aDNA) studies have shown that HBV was already endemic in WHG and Neolithic populations [63, 64], albeit in form of phylogenetically distinct strains. The occurrence of frequent protective HLA class II alleles (though different ones) suggests that the virus may have been a strong selective pressure in both WHG and farmers.

An additional factor contributing to the HLA shifts between EF and LF may be changing pathogen landscapes during the Neolithic. It has been hypothesized that the adoption of the Neolithic lifestyle (e.g., sedentary groups living closely with domesticated animals) was associated with an increase in infectious diseases and epidemics [65]. However, archeological and aDNA studies including thousands of samples have so far not provided evidence for large-scale epidemics, only infections caused by a limited number of pathogens including HBV [63, 64, 66–68]. Based on current data, this low pathogen load did probably not change from the Early to the Late Neolithic but increased from the Bronze Age onwards [68]. This is consistent with findings from ancient populations showing that genetic adaptation of humans to infectious diseases mainly occurred after the beginning of the Bronze Age [69].

With regard to the HLA allele repertoire, it is noteworthy that EF and LF often had lower diversity compared to modern populations (Fig. 4). This means that a few alleles were observed at exceptionally high frequencies (> 20%) (Additional file 1: Data S10; Additional file 2: Fig. S13). Theory predicts that the presence of such common HLA alleles over extended periods of time should increase the probability that pathogens evolve evasion mutations to reduce the likelihood of their recognition by the immune system [70]. The maintenance of the frequent alleles over two millennia might therefore support the observation that the low pathogen threat and load likely remained the same throughout the Neolithic.

When comparing the two Neolithic groups with modern Germans, we observed significant changes in the frequencies of 17 HLA alleles (Additional file 2: Table S4). Notably, 14 of these alleles exhibited a significant decrease in frequency over time. The most pronounced shifts were observed in HLA class II alleles, but substantial changes were also detected in HLA class I alleles such as HLA-B*27:05 and HLA-B*51:01. These alleles are strongly associated with inflammatory diseases including ankylosing spondylitis (AS), psoriatic arthritis, and Behçet’s disease today [71–74]. The elevated frequencies of HLA-B*27:05 and HLA-B*51:01 in Neolithic populations have been reported previously [6, 15]. Interestingly, one case of AS has been described for a Late Neolithic individual [75] and several others from various Medieval sites in Europe [76–79]. These observations suggest that mismatch diseases like AS affected already pre-modern populations. They also raise the possibility that ancient individuals could have suffered from other chronic inflammatory disorders (not visible on bone) that are usually considered modern diseases. The Neolithic transition may already have provided a mismatch between lifestyle and genetics. Given the negative impact of such diseases on overall fitness, it is plausible that negative selection might have acted on the associated alleles. Supporting this, the proxy SNP-alleles for both HLA-B*27:05 (rs13202464-G) and HLA-B*51:01 (rs4947296-C) have declined in frequency since the Neolithic [6] and exhibit signs of negative selection [80]. Conversely, some alleles outside the HLA region that have anti-inflammatory effects showed evidence of positive selection (e.g., rs11209026 in the IL23R gene [81]) at the onset of the Neolithic.

Surprisingly, seven HLA alleles present at high frequencies (≥ 10%) in modern Germany were apparently absent in both EF and LF (Additional file 2: Table S5). This finding suggests that these common HLA alleles were likely introduced after the Neolithic through admixture, possibly with groups carrying steppe-related ancestry. For example, the DRB1*15:01 allele, an important risk factor for multiple sclerosis, was introduced into the European gene pool by steppe herders and maintained afterwards [6, 14]. The same trend appears to apply to DQB1*06:02 that forms a haplotype with DRB1*15:01 [82] and was also not present in the Neolithic (Additional file 2: Table S5). Even more striking is the case of DQB1*02:01, an allele linked to celiac disease today [83], which was also absent in EF and LF, but has increased to a frequency of 20% in modern populations over the last 4000 years [84]. This increase seems counter-intuitive, given that a gluten-rich diet (e.g., cereals) was common during this period, raising the question of whether individuals carrying the allele would have been adversely affected by celiac disease, potentially impairing their fitness [85]. For both alleles DRB1*15:01 and DQB1*02:01, it has been proposed that their frequency increase may be a result of pathogen-driven selective pressures [14, 85].

## Conclusions

By expanding the genetic data for WBC-associated populations, we confirmed that they represent a distinct group characterized by a high WHG ancestry proportion and a dominance of Y-chromosome haplogroup I, with site-specific sub-lineages. This WHG ancestry was introduced through male-biased admixture, probably due to increased societal permeability and mobility. Notably, the WHG introgression also led to significant changes in the HLA profile from EF to LF. The limited diversity observed in certain HLA loci in the Neolithic groups, compared to modern populations, supports the hypothesis of a low pathogen load during that time. The Neolithic transition may already have introduced a mismatch between genetics and lifestyle, reflected in the frequency decline of alleles associated with chronic inflammatory diseases (e.g., HLA-B*27:05). These alleles could have been subject to negative selection due to their deleterious effects. Future research based on true HLA genotypes from WHG, steppe and Bronze Age populations will be critical for clarifying the evolutionary processes that shaped HLA diversity before and after the Neolithic.

## Methods

### Sampling

In total, we collected petrous bones and/or teeth from 175 individuals from six sites within Germany ranging from the Early to the Late Neolithic (Fig. 1A, Table 1, Additional file 1: Data S1).

### DNA extraction and library preparation

DNA was extracted from teeth and/or petrous bones of all individuals and converted into partial uracil-DNA glycosylase (UDG) libraries [86] following established laboratory guidelines for aDNA work [87]. Shotgun sequencing was performed on the Illumina HiSeq 6000 (2 × 100) platform of the Institute of Clinical Molecular Biology in Kiel. Additionally, UDG-treated libraries were enriched for the HLA region applying a custom bait capture designed by Wittig et al. [88]. The targeted HLA capture was successful for 112 samples (Additional file 1: Data S1).

### Mapping

The removal of adapter sequences as well as the merging of paired-end reads was performed with ClipAndMerge [89] v1.7.7. Mapping to both the human genome (build hg19) and human mt genome references was done with BWA aln [90] v0.7.15 using reduced mapping stringency settings (flag -n 0.01) to account for mismatches expected in aDNA. Duplicates were removed with DeDup [89] v0.12.1.

### Contamination estimation and genetic sex determination

To evaluate the ancient authenticity of the extracted DNA, we assessed the terminal damage of reads by calculating the frequency of C to T substitutions with DamageProfiler [91] v1.1. After validation, the first two positions from the 5′ and 3′-ends of the reads were removed with bamUtil [92] v1.0.15. mtDNA contamination was estimated by analyzing sequence deamination patterns and fragment length distributions with Schmutzi [93] v1.5.5.5. Additionally, contamination in male samples was measured by assessing X-chromosome heterozygosity with ANGSD [94] v0.935. Samples that showed more than 5% mtDNA or X-chromosome contamination were excluded from further analysis. In cases where contamination estimation with Schmutzi was not possible, the placement of the individuals in the PCA plot was additionally used to assess if the samples should be excluded. Sex was genetically determined by considering the ratio of sequences aligning to the X-chromosome and autosomes [95]. Only samples with more than 1000 reads were considered for sex determination.

### Genotyping

SequenceTools [96] v1.2.2 was used to generate pseudo-haploid genotypes on 1,233,013 SNP positions [5, 53, 97]. Samples with fewer than 20,000 genotyped SNPs were excluded from the analysis.

### Inclusion of Niedertiefenbach data

In addition to the datasets generated for individuals from the six sites, we incorporated data from the previously published Niedertiefenbach population [6]. Niedertiefenbach was included in all subsequent analyses, with the exception of kinship inference and metagenomic pathogen screening, the results of which have already been reported [6].

### Principal component analysis

The genotyped samples in this study were merged with both the 1240 K and Human Origins (HO) panels of the Allen Ancient DNA Resource (AADR; v50.0.p1), containing previously published genotypes of ancient and modern individuals [98]. Unless stated otherwise, the 1240 K panel was used for all analyses described in the “Methods” section. The PCA was performed with *smartpca* v16000 [99] from the EIGENSOFT package v7.2.1 with the “lsqproject” option and 597,573 SNPs (HO panel). The calculation of principal components was based on a subset of 64 modern populations (*n* = 1154 individuals; Additional file 1: Data S13) from West-Eurasia, while the remaining individuals from the merged dataset were projected into that space.

### Outgroup f3 statistics

Shared genetic drift was calculated with the program *qp3Pop* v650 from the Admixtools package [100] v7.0.1 in the format f_3_ (sample population, test population; Mbuti), where “sample population” refers to the seven populations in this study and “test population” refers to published ancient groups available in the merged dataset (groups used are listed in Additional file 1: Data S14). Only results with a SNP overlap exceeding 20,000 were used for the plots.

### Admixture analyses

First, we performed unsupervised admixture analysis to explore the population structure of the seven populations in a hypothesis-free manner. For this analysis, we selected a subset of 295 published populations (*n* = 1405 individuals) mostly representing the prehistoric genetic diversity in Eurasia (see Additional file 1: Data S15 for a comprehensive list of samples). The dataset was then filtered by MAF (command “–maf 0.01”) and pruned in PLINK [101] v1.90b6.21, with an r^2^ threshold of 0.4, a window size of 200 and a step size of 25 (command “–indep-pairwise 200 25 0.4”). The unsupervised admixture analysis was performed with the software ADMIXTURE v1.3.0 [102] using a range of 2 to 12 components (K) with 100 bootstraps each.

Next, we employed a supervised modelling approach with qpWave v1200 and qpAdm v1201 from the Admixtools package [100] v7.0.1. qpWave was used to test the minimum number of source populations that could explain the genetic composition of the s even populations studied here, while qpAdm was used to quantify ancestry proportions. We tested specific models deemed most archeologically plausible and relevant to our target populations. The list of source populations and outgroups used for each analysis is available in Additional file 1: Data S3-6. The qpAdm and qpWave programs were run with the option “allsnps: NO.” To provide an overall estimate of the general WHG and early European Neolithic farmer ancestries across several qpAdm models, we averaged the estimates from feasible models (*p* ≥ 0.05) where the proportions fell within the [0,1] range.

Following the qpWave and qpAdm analyses, we used DATES [103] v753 to estimate the most recent time of admixture in each of our reported LF populations, with the parameters 'binsize: 0.001’, 'maxdis: 1.0’, 'seed: 77’, 'jackknife: YES’, 'qbin: 10’, 'runfit: YES’, 'afffit: YES’, 'lovalfit: 0.45’, 'minparentcount: 1’. A generation time of 29 years was used to calculate the admixture calendar years [104]. The source combinations used for DATES are listed in Additional file 1: Data S5.

Additionally, to explore how the WHG ancestry in published groups from the region changed over time, particularly in comparison to our populations, we conducted a supervised ADMIXTURE analysis. This analysis included published samples dating from the Early Neolithic to the Bronze Age (*n* = 175 groups, *n* = 800 samples) and utilized three ancestral sources—WHG, early European Neolithic farmers, and steppe herders—to account for this broad temporal range. The list of individuals included in the analysis, along with the populations used as proxies for these ancestral components, is provided in Additional file 1: Data S16. Before conducting the analysis, the dataset was pruned as described above for the unsupervised ADMIXTURE analysis.

### Sex-biased admixture in late farmers

To assess sex-biased admixture in the LF populations (Altendorf, Warburg, Rimbeck, Niedertiefenbach), we first employed the Q statistic [105–107], which quantifies the relative genetic drift between the X-chromosome and autosomes as measured by F_st_. Under a model with equal effective population sizes for males and females, Q is expected to be 3/4, as there are three X-chromosomes for every four autosomes in the population. Deviations from this value could indicate sex-biased demographic processes. As an initial step, we estimated genetic differentiation on the X-chromosome (F_st_X) and autosomes (F_st_A) between LF and the two source populations, WHG and AN. For this, we used the SNPs of the 1240 K panel and filtered out positions with *r*^2^ > 0.4 (plink command “indep-pairwise 200 25 0.4”). SNPs in the pseudoautosomal regions of the X-chromosome were also removed. After filtering, 560,930 autosomal and 5004 X-chromosomal SNPs remained. Individuals with fewer than 1000 SNPs covered on the X-chromosome were removed from the analysis. We then used the Weir and Cockerham weighted F_st_ values obtained with plink (command “–fst”) to calculate the statistic Q as

$$Q=\frac{ln\left(1-2{F}_{st}A\right)}{ln\left(1-2{F}_{st}X\right)}$$

As Q can be influenced by factors other than sex-biased admixture [105–107], we also computed the amount of WHG ancestry on the X-chromosome versus autosomes using supervised ADMIXTURE analyses with WHG and AN as sources (populations used as proxies for these ancestries are described in Additional file 1: Data S16). As the number of SNPs available for the analyses on the X-chromosome is low (*n* = 5004) compared to autosomes (*n* = 560,930), we down-sampled autosomal SNPs 1000 times relative to the number of SNPs available for the X. For each individual, we calculated the ratio of WHG ancestry on the X-chromosome versus autosomes. We used the Wilcoxon signed-rank test to assess significant differences between the means of X and autosomal WHG ancestry [105]. To investigate the robustness of the admixture estimates on the X-chromosome given its low coverage, we selected a set of 15 individuals with relatively high X-chromosome coverage (i.e., > 4000 SNPs). Then, we systematically down-sampled the X-chromosome data to 1000 SNPs, repeating this process 1000 times. Using supervised ADMIXTURE, we generated an empirical distribution of ancestry estimates from the down-sampled data. Finally, we compared these estimates to those derived from the full set of SNPs, calculating the *r*^2^ statistic to assess the consistency and robustness of the ancestry estimates (Additional file 2: Fig. S15).

### Mitochondrial and Y-chromosome haplogroups

mtDNA haplogroups were determined with HaploGrep2 [108] v2.4.0 and Y-haplogroups with yHaplo [109] v1.1.2. A mapping and base quality threshold of 20 was used. For mtDNA, only haplogroups with a quality score > 0.8 were considered. For Y-haplogroups, the presence of at least 10 derived alleles was used as a threshold to make a call.

### Kinship analysis

For the population of Niedertiefenbach, kinship analysis has already been done previously [6]. For the other six populations studied here, we estimated kinship using the method described in Fowler et al. [110]. Shortly, for each pair of individuals, we calculated pairwise allelic mismatch rates in autosomal sites of the 1240 K panel. We then computed relatedness coefficients *r* for each pair using the formula

$$r=1-\left(\frac{2\left(x-\left(\frac{b}{2}\right)\right)}{b}\right)$$

where *x* is the mismatch rate of the pair of individuals and *b* the expected mismatch rate for two unrelated individuals from the same population. To calculate the constant *b*, we first merged data from our six populations (*n* = 83 individuals) with published data from 15 Neolithic populations located in present-day Germany (*n* = 155 individuals). Then, we calculated pairwise mismatch rates for all combinations of two individuals from the merged dataset (28,203 comparisons) and used 1000 bootstrap samples to calculate 95% confidence intervals. We filtered out pairwise comparisons with fewer than 100 K overlapping SNPs (7101 comparisons remained after filtering) and calculated *b* as the median mismatch rate of the filtered dataset (*b* = 0.2593), a value similar to that obtained by Fowler et al. [110] (0.2504) using Neolithic individuals from England. We then applied our obtained value of *b* in the formula described above to calculate the relatedness coefficient for each pair of individuals. Relationship degrees were annotated using the same cutoffs as in Fowler et al. [110], but as a conservative approach, only kinships of 1st and 2nd degrees were considered. Pairwise comparisons with fewer than 2500 overlapping SNPs or with a large confidence interval leading to annotation of more than 2 possible degrees of kinship were not considered. mtDNA and Y-chromosome haplogroups, when available, were also considered in assessing kinship.

### Runs of homozygosity (ROH)

We screened for ROH using HapROH [49] v0.3a4 with the default parameters. Only samples with more than 400,000 SNPs genotyped from the 1240 K panel were included. The results were merged with previously published ROH estimations [49]. Due to the small sample size of EF, seven published populations were added for calculating the average sum of ROH for this group (Additional file 1: Data S8). We did not merge our LF samples with other published Late Neolithic populations, as our focus was specifically on WBC-associated individuals. The ROH results were then used to infer the effective population size (N_e_) also with HapROH using the default parameters. A PERMANOVA test using 99,999 permutations was performed with the python3 module skbio v0.5.6 to test for significant differences in the average sum of ROH between groups.

### Imputation and chromosome painting

To test whether the high WHG ancestry in our LF also influenced their HLA allele pool, we conducted local ancestry inference with RFMIX [111] v2.03-r0. We first selected well-covered LF as well as published WHG and AN individuals (mean depth ≥ 0.5X and breadth of coverage ≥ 50%) for imputation. The selected samples were imputed and phased with GLIMPSE [112] v2.0.0 using the 1000 Genomes dataset as reference panel. We filtered the resulting VCF to include only positions within the strict accessibility mask from the 1000 Genomes Project [113] and excluded regions annotated in the RepeatMasker track from the UCSC browser [114]. Additionally, we filtered out variants with a MAF < 0.05, INFO score < 0.9, or mean genotype probability (GP) < 0.99. We also excluded individuals with a mean GP < 0.99. The final dataset for chromosome painting comprised 39 samples (LF = 19, WHG = 10, AN = 10; listed in Additional file 1: Data S11) and 417,784 SNPs. The local ancestry inference in LF individuals was conducted using AN and WHG as source populations with RFMix v2.03-r0, employing the additional parameters "-e 10 –reanalyze-reference." The RFMix analysis also required specifying the average number of generations since the expected admixture (via the "-G" parameter). We set this parameter to 22 generations, based on the average of estimates calculated across various models tested with DATES (Additional file 1: Data S5). Using the output from RFMix that provides the probability of each ancestry for each SNP and haplotype, we calculated the mean probability of the WHG origin for each SNP across all individuals and referred to it as “mean WHG ancestry.” We then computed a *Z*-score for each SNP* i* as follows:

$${Z}_{i}=\frac{{A}_{i}-\mu }{\sigma }$$

where *A*_*i*_ represents the mean ancestry painting for a SNP *i*, μ is the genome-wide mean, and σ is the genome-wide standard deviation.

### HLA genotyping and frequency calculations

Genotyping of the HLA alleles was performed using the TARGT v2 pipeline (Targeted Analysis of sequencing Reads for GenoTyping) [115] and OptiType v1.2.1 [116]. TARGT was especially designed for the analysis of low-coverage sequences and was shown to yield reliable HLA class I and II calls with aDNA [115]. To ensure a higher reliability of the results, the genotyping by TARGT was done by two independent scientists. OptiType can only perform typing of HLA class I alleles [116]. A recent benchmarking study revealed that its performance with low-coverage data is better than other automated algorithms [117]. We have successfully used both methods for typing HLA alleles in previous aDNA studies [6, 118, 119]. For the three HLA class I loci (HLA-A, -B, and -C), we applied here a combination of both TARGT and OptiType and cross-checked the genotyping results. Discordant calls were excluded from the analysis. For the three HLA class II loci (HLA-DPB1, -DQB1, and -DRB1), genotyping was done with TARGT only. All analyses were performed at two-field HLA allele resolution. For the allele frequency calculations, we grouped the populations according to their dates, cultural affiliations, and population structures as EF (Niederpöring, Fellbach-Oeffingen, and Trebur) and LF (Altendorf, Rimbeck, Warburg, and Niedertiefenbach). We excluded from the allele frequency calculations seven individuals from seven kinship clusters containing 1st degree relationships (Altendorf = 2, Fellbach-Oeffingen = 1, Trebur/Hinkelstein = 4). For Niedertiefenbach, we included HLA profiles of 56 unrelated individuals in the LF group, 33 of which were generated as part of this study and 23 of which were previously published [6]. This addition increased our data set to 135 individuals in total (EF = 45; LF = 90; Additional file 1: Data S1). For 22 individuals, the targeted HLA capture was successful, but no shotgun data of sufficient quality was available to allow population genetic analysis. However, both the archeological context and aDNA damage plots, which showed distinct deamination patterns, demonstrated the ancient origin of the samples used (Additional file 1: Data S2) and thus these were kept for the HLA frequency calculations. For comparison with modern Germans (*n* = 3,456,066 [50], data from the Allele Frequency Net Database [120] were accessed. We used Fisher’s exact test from the python3 module scipy v1.13.1 to assess whether the observed allele frequencies between groups were significantly different. The *p*-values were corrected for multiple testing with the two-stage Benjamini and Hochberg (TSBH-FDR) procedure using the python3 module statsmodels v0.13.5. Additionally, to confirm that the alleles that were found to be significantly different using the previous approach were not due to different population sizes, we also applied down-sampling. More precisely, we generated 100 resamples with replacement, each of size *n*, where *n* corresponds to the smallest population size. The frequency distributions from these resamples were then compared in a pairwise manner between the groups using a two-sided Mann–Whitney *U* test from the python3 module scipy v1.13.1. The *p*-values were also corrected for multiple testing using TSBH-FDR. Frequencies of the possible haplotypes DRB1*13:01-DQB1*06:03 and DRB1*08:01-DQB1*04:02 were calculated using the expectation–maximization algorithm implemented in the Arlequin v3.5 software [121].

In order to investigate the influence of admixture between EF and WHG on the HLA allele pool, we used the local ancestry estimations for 19 LF individuals (see section “Imputation and chromosome painting”). First, we identified three alleles with significant increase in frequency from EF to LF (Fig. 2). For each allele, we divided the 19 LF individuals into carriers and non-carriers and calculated the mean WHG ancestry per individual across the MHC region. Next, we compared the mean WHG ancestry between the two groups using the Mann–Whitney test with Holm-Sidak correction.

Shannon’s diversity index (H’) was calculated by using the diversity function from the *vegan* v2.6.4 R package to measure the genetic diversity of HLA alleles in Neolithic and modern populations. Five populations with European ancestry from the 1,000 Genomes Project [52], namely British from England and Scotland (GBR), Finnish in Finland (FIN), Iberian populations in Spain (IBS), Toscani in Italy (TSI), and Utah residents (CEPH) with northern and western European ancestry (CEU) were included in the analysis to obtain a better estimation of the modern HLA diversity. We used a down-sampling approach to control for differences in sample sizes between ancient and modern populations, since Shannon’s diversity index uses proportions of alleles which can be affected by sample sizes. Specifically, for each locus, we first identified the population with the smallest sample size (*n*), which was always EF. Then, for each population, we generated 100 random samples with replacement and size *n* using the sample function in R (v4.3) and calculated Shannon’s diversity index. This resampling with replacement approach allows each individual to be selected multiple times within each sample. The probability of sampling an allele was weighted by its frequency within each population. This approach allowed us to obtain a distribution of Shannon’s diversity index values for each population including EF. We compared the distribution of Shannon’s diversity index values between populations using the Kruskal–Wallis and Dunn’s tests.

### Metagenomic screening

The sequencing reads were screened for the presence of pathogens following an in-house pipeline [122, 123] using MALT [124] v0.4.1 with a semi-global alignment mode and a minimum percent identity of 90% to align the samples against a database of 27,730 bacterial and 10,543 viral complete genomes [125, 126].

## Supplementary Information

Additional file 1. Supplementary Data

Additional file 2. Supplementary Notes, Figures and Tables

Additional file 3. Review history

## Data Availability

Aligned sequencing reads for samples reported in this study are available from the European Nucleotide Archive (ENA), accession no: PRJEB53796. Sequencing reads for the previously published Niedertiefenbach samples are available under accession no: PRJEB35327. Scripts to replicate the results from the main figures are available under MIT license on GitHub [127]. We have deposited the version of the code used in the manuscript on Zenodo [128].
