# Supplementary material for: Admixture as a source for HLA variation in Neolithic European farming communities
Source: Genome Biol. 2025 Feb 28;26:43. doi: 10.1186/s13059-025-03509-6 (PMC11869582; doi:10.1186/s13059-025-03509-6)
Supplement: Supplementary file 2 — Additional file 2. Supplementary Notes, Figures and Tables [file 13059_2025_3509_MOESM2_ESM.pdf]

Supplementary Information for:

Admixture as a source for HLA variation in Neolithic European farming communities

da Silva et al.
*Corresponding author contact information: b.krause-kyora@ikmb.uni-kiel.de

This PDF file includes:

Note S1
Figures S1 - S15
Tables S1 -S5

Other supplementary materials for this manuscript include the following:

Data S1 - S16

Note S1: Archaeological context information

Altendorf

The gallery grave of Altendorf in Hesse, Germany, is situated 24 km southwest of Kassel in the West Hesse foothills. It was discovered in 1907 and excavated in 1934. The grave contained the remains of at least 235 individuals, along with grave goods such as pottery, bone and copper artifacts. The typological classification of these items indicates that the grave was used during the transition from the Late Neolithic Wartberg culture (WBC) to the Final Neolithic Corded Ware culture in the late fourth millennium BCE. Anthropological and archaeological evidence suggests that the tomb was used over several generations. References in the main text: [114-118].

Fellbach-Oeffingen

Oeffingen is a suburb of the town of Fellbach in Baden-Württemberg, Germany. On the western outskirts of Oeffingen, extensive Neolithic settlement remains were discovered, predominantly dating to the Linear Pottery culture. Between 1986 and 1987, 110 graves were excavated in an area of 50 by 40 meters. The excavation likely covered the entire burial ground. The graves were randomly distributed but predominantly oriented SE-NW, with skulls positioned in both directions. Among the graves, 102 were inhumation burials in a crouched position. Due to the loess loam soil, bone preservation was poor. In 15 cases, cremated remains were scattered over the skeletons in the grave pits, while eight additional graves appear to be cremation-only burials. Artifacts were scarce, with only 35 graves containing items such as axes, vessels and bone tools. References in the main text: [104,105].

Niederpöring

Niederpöring is a part of the municipality of Oberpöring in Bavaria (Germany). In 1983, a large area along the Isar river was archaeologically investigated in Niederpöring. Among the numerous prehistoric features identified were earthworks from the Linear Pottery and Münchshöfen cultures, along with a Linear Pottery settlement and seven graves. The Linear Pottery burial group was located directly south of the terrace edge. While the boundaries were clearly identified to the west, south, and east, they were not fully traced to the north, suggesting that some graves may have been missed. All seven burials were placed individually in specially constructed grave pits, most of them spaced several meters apart without any discernible spatial order. Each burial was an inhumation, with the deceased laid in a crouched position. Five of the seven graves contained grave goods, primarily rich bead jewelry, but no pottery, making typological dating within the Linear Pottery culture impossible. Radiocarbon dating of human bones from the site placed the burials between 5205 and 4916 cal BC. Combined with finds from nearby settlements, these dates point to the late phase of the Linear Pottery culture. Reference in the main text: [106].

Rimbeck

The gallery grave of Rimbeck is situated on a limestone hilltop north of the Diemel Valley, on the edge of the Warburger Börde, at the outskirts of today’s Warburg-Rimbeck, North Rhine-Westphalia, Germany. The grave is on the southeastern edge of an older dating ditch system. Prospections of the

area also revealed a Younger Neolithic settlement on the northeastern edge of the plateau. The gallery grave was first excavated in 1906/1907 and re-examined in 1985/1986. Artefacts recovered from the grave, including bone and stone arrowheads, flint tools, pottery (notably a well-preserved Rimbeck-type mug), and animal tooth jewelry, link the burial to the Wartberg culture. The human remains analysed in this study were found during the re-examination. Radiocarbon dating places the burial in the late 4th and early 3rd millennium BC. References in the main text: [119, 127-129].

Trebur

The Trebur site is located in the Northern Neckar Ried, within the Hessian Rhine Plain. As part of the Upper Rhine Lowlands, the Hessian Rhine Plain was densely populated during the Neolithic period. A total of 134 graves were excavated at the site. Based on the grave goods, the burials can be associated with two archaeological groups: Hinkelstein and Großgartach. The site's exceptional preservation, compared to other locations in the region, and the presence of these two groups within one cemetery have sparked extensive discussions on chronological and social differentiation. The individuals were buried in a stretched position, with their heads facing southeast, although half of the Großgartach graves were oriented in the opposite direction. Most of the graves are arranged in rows, with individual groups separated by distance or slight variations in orientation. Isotope analyses revealed differences between males and females, but no significant differences between the two groups, Hinkelstein and Großgartach. The debate about the site's precise chronology continues, with some scholars suggesting that the pottery groups follow a sequential order, while others argue for the partial contemporaneity of the two groups. References in the main text: [107-113].

Warburg

Between 1986 and 1993, a collective burial necropolis consisting of four gallery graves and a wooden chamber was investigated on the northwestern outskirts of present-day Warburg, North Rhine-Westphalia, Germany. Three of the gallery tombs had entrances on the narrow side (Züschen tomb type), while one gallery tomb had an entrance on the long side (Rimbeck type). A wooden chamber (Warburg II) was also uncovered, featuring a rectangular layout of 8.8 × 4.9 meters, including an anteroom and a main chamber. The main chamber was characterized by foundation trenches up to 1 meter deep and three post holes. However, the interpretation of this structure as a grave remains uncertain. Radiocarbon dating of the gallery tombs has yet to fully clarify whether the tombs were used simultaneously or sequentially. The dates suggest the tombs were in use from around 3400 cal BC until the later Wartberg period, approximately 3000 cal BC. References in the main text: [119-126].

In this study, individuals from gallery graves III and IV were analyzed:

- **Gallery grave III**: The minimum number of individuals was 80, including at least 29 children and 48 adults. Twenty-one skeletal complexes were found, some of which allowed the reconstruction of burial sequences. Six skeletons were oriented N-S and thirteen S-N, with five in a supine position and three in a crouched position. Arms were often bent over the upper body or resting at the pelvis.
- **Gallery grave IV**: The minimum number of individuals was 65, including at least 13 children and 38 adults. Burial positions suggest a division, with more women buried in the northern part

and more men in the southern part. Burial sequences could be identified for 13 skeletons. Most were found in supine positions, though one prone burial was recorded. Seven individuals were in a stretched position and four in a crouched position, with head orientations varying between north (six instances), south (five), and west (three). Arm positions were angled, with hands on the shoulder or chest.

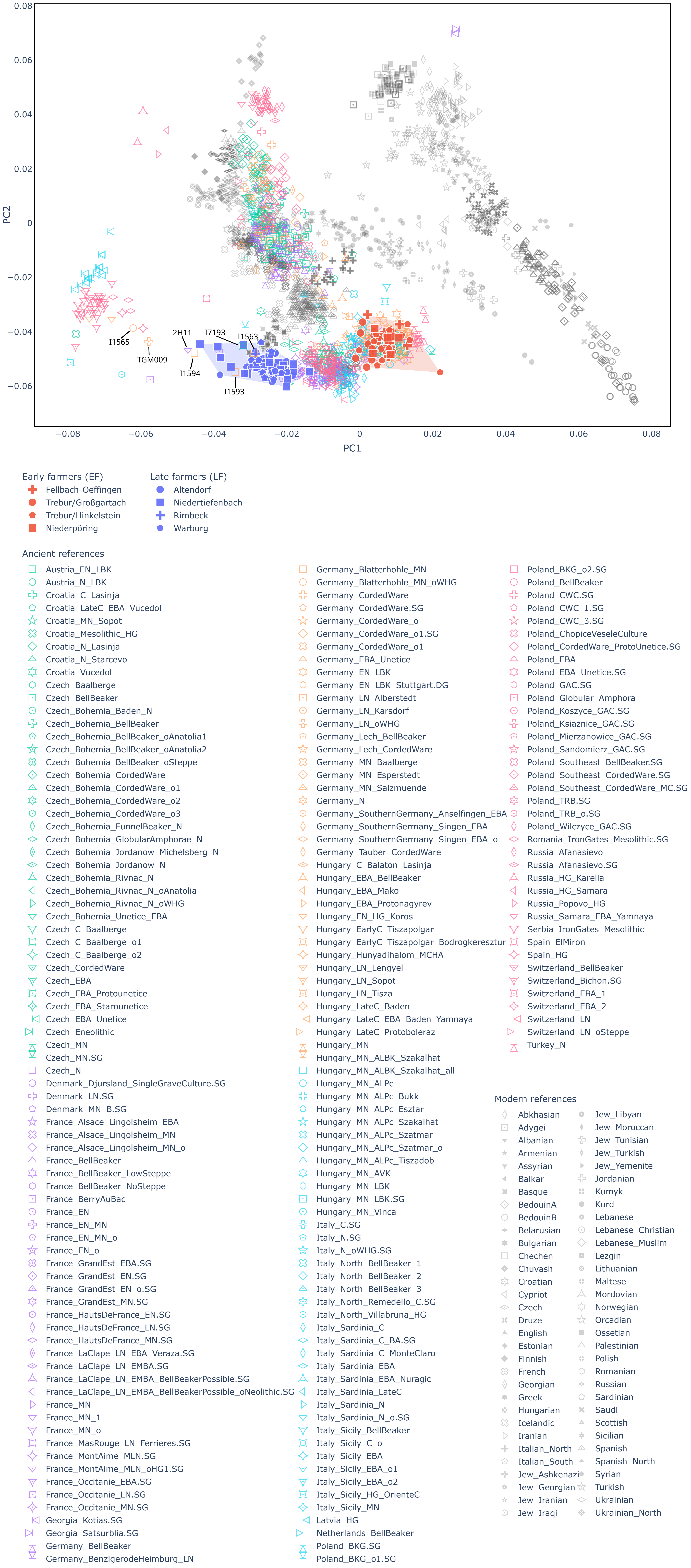

Fig S1. Principal component analysis with modern samples shown. The ancient individuals were projected onto modern West-Eurasian variation. Convex hulls highlight the space filled by early farmers (EF) and late farmers (LF). Publicly available data from Mesolithic, Neolithic and Bronze Age populations are also included. Individuals mentioned in the main text are labeled.

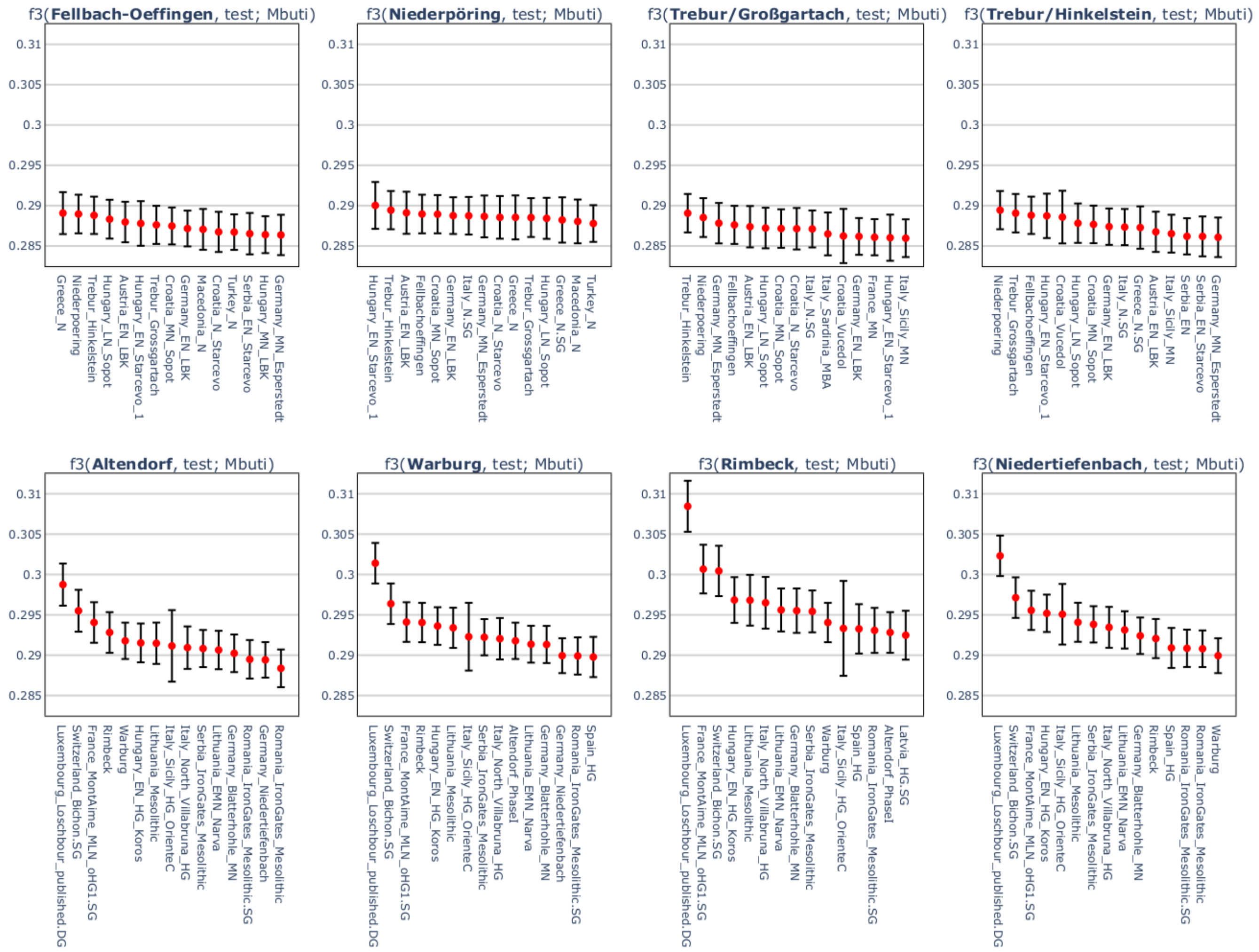

Fig S2. Outgroup f3 statistics. The f3 statistics were calculated in the form f3(pop, test; Mbuti) to measure the shared genetic drift between each of the populations that are the focus of this study (denoted as "pop") and other published ancient populations (denoted as "test"). The 15 test populations with the highest f3 scores from each test are displayed.

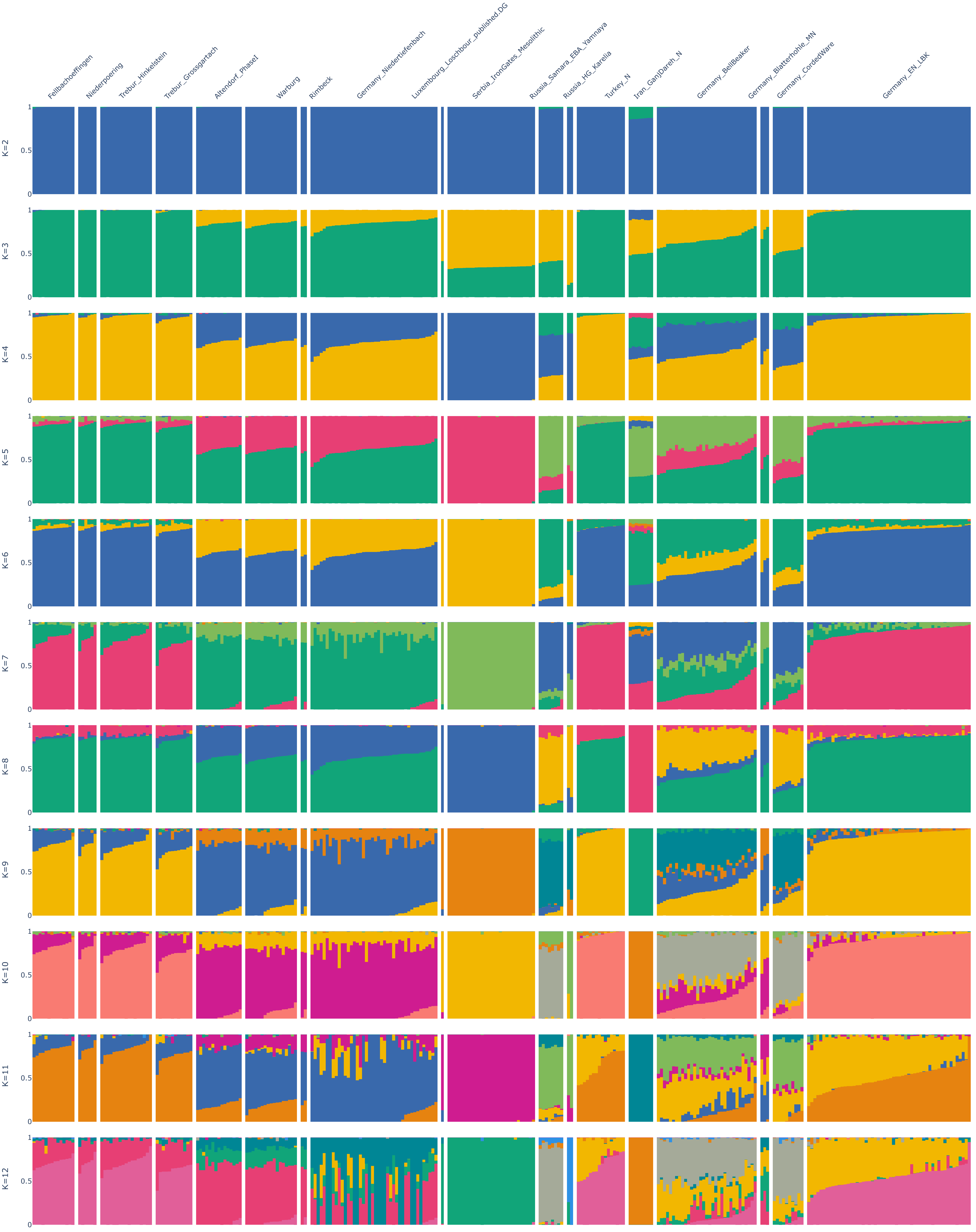

Fig S3. Results from unsupervised admixture. The analysis was conducted using two to twelve components (K) across 295 selected ancient and modern populations/individuals, with 11 representative ones plotted to reflect typical WHG, steppe, and Anatolian farmer ancestries. Each K was run with 100 bootstraps. HG = Hunter-gatherer; N = Neolithic; EN = Early Neolithic; MN = Middle Neolithic; EBA = Early Bronze Age; LBK = Linear Pottery culture.

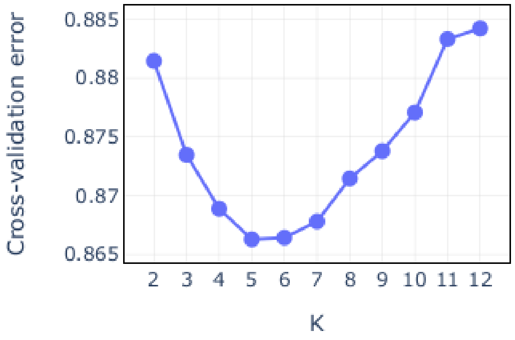

Fig S4. Cross-validation error for unsupervised admixture analysis. The cross-validation error was calculated for each value of K, representing the number of components modeled. Lower cross-validation error values indicate a better fit to the data.

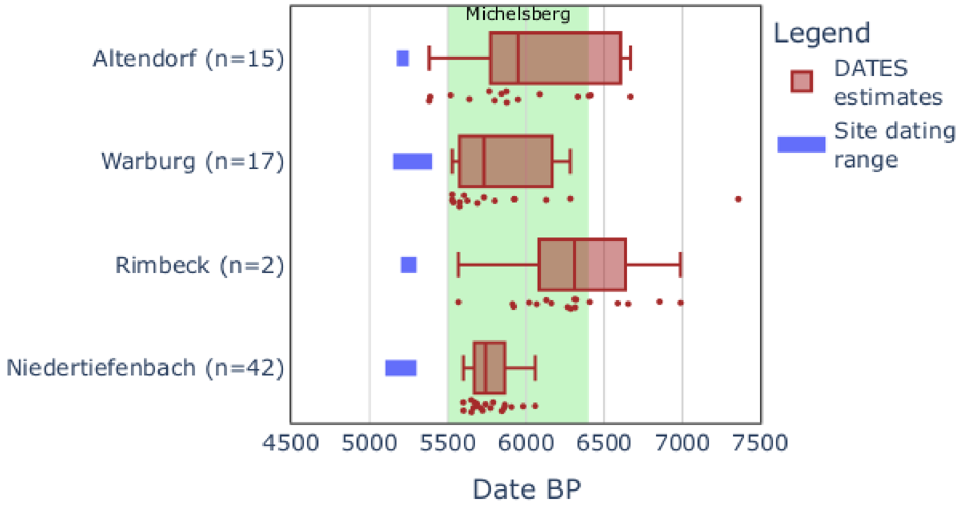

Fig S5. Inference of time of admixture with DATES. The admixture dates for late farmer (LF) populations were modeled using various potential source groups. Results for each model are represented by the brown markers, while the boxplots summarize the distribution of the admixture dates across all models. Results from infeasible models (mean generation time < 0) and models where the estimated admixture date overlaps with the site's dating range are excluded from the plots. The sample sizes (n) for each group are provided in parentheses next to the corresponding site name. The period associated with the Michelsberg culture is highlighted in green.

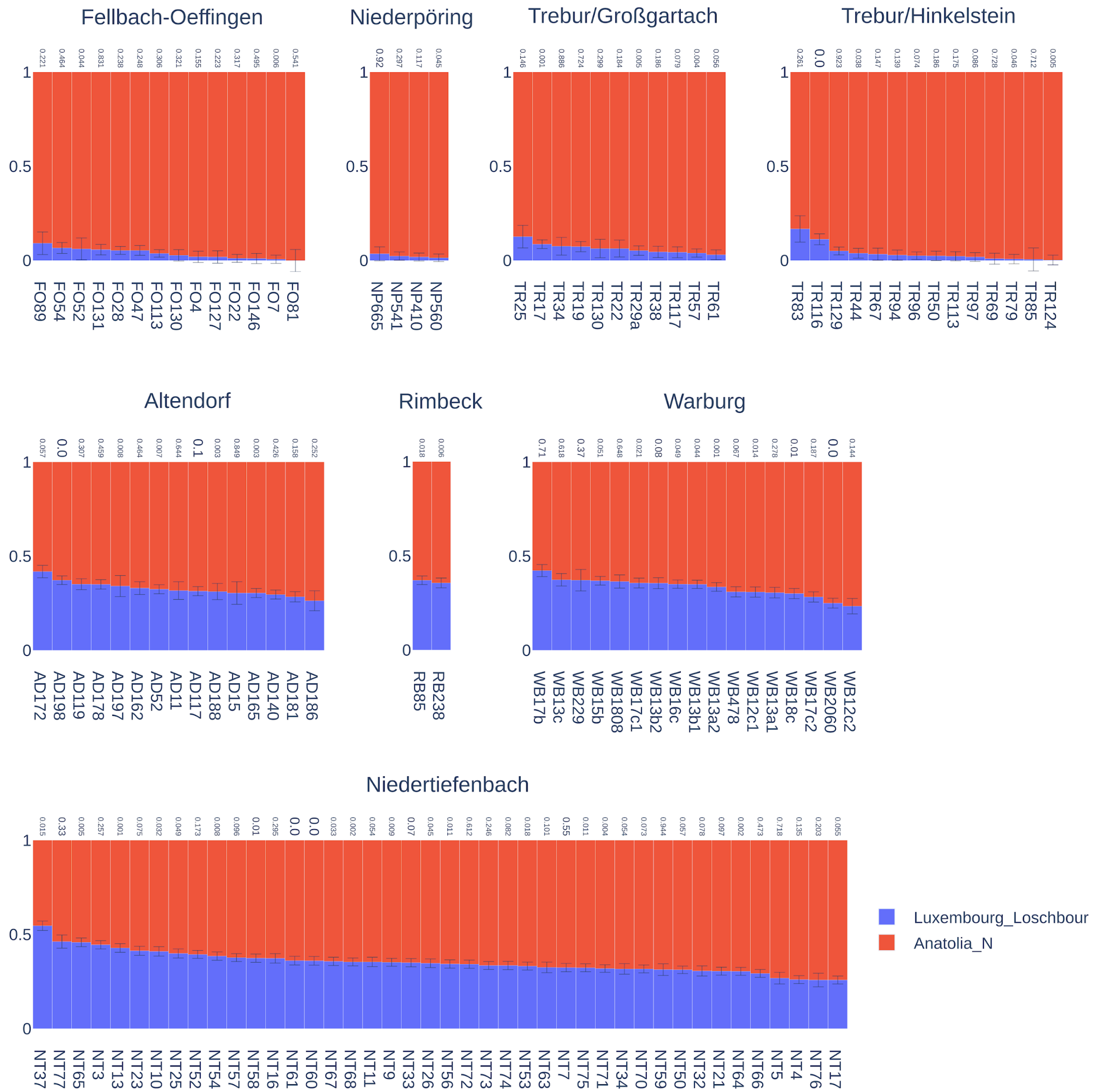

Fig S6. Individual qpAdm-based admixture models using two sources (Anatolian farmers and western hunter-gatherer from Luxembourg Loschbour). Only individuals with feasible models are shown. The values on top of the stacked bars refer to the p-value of each model.

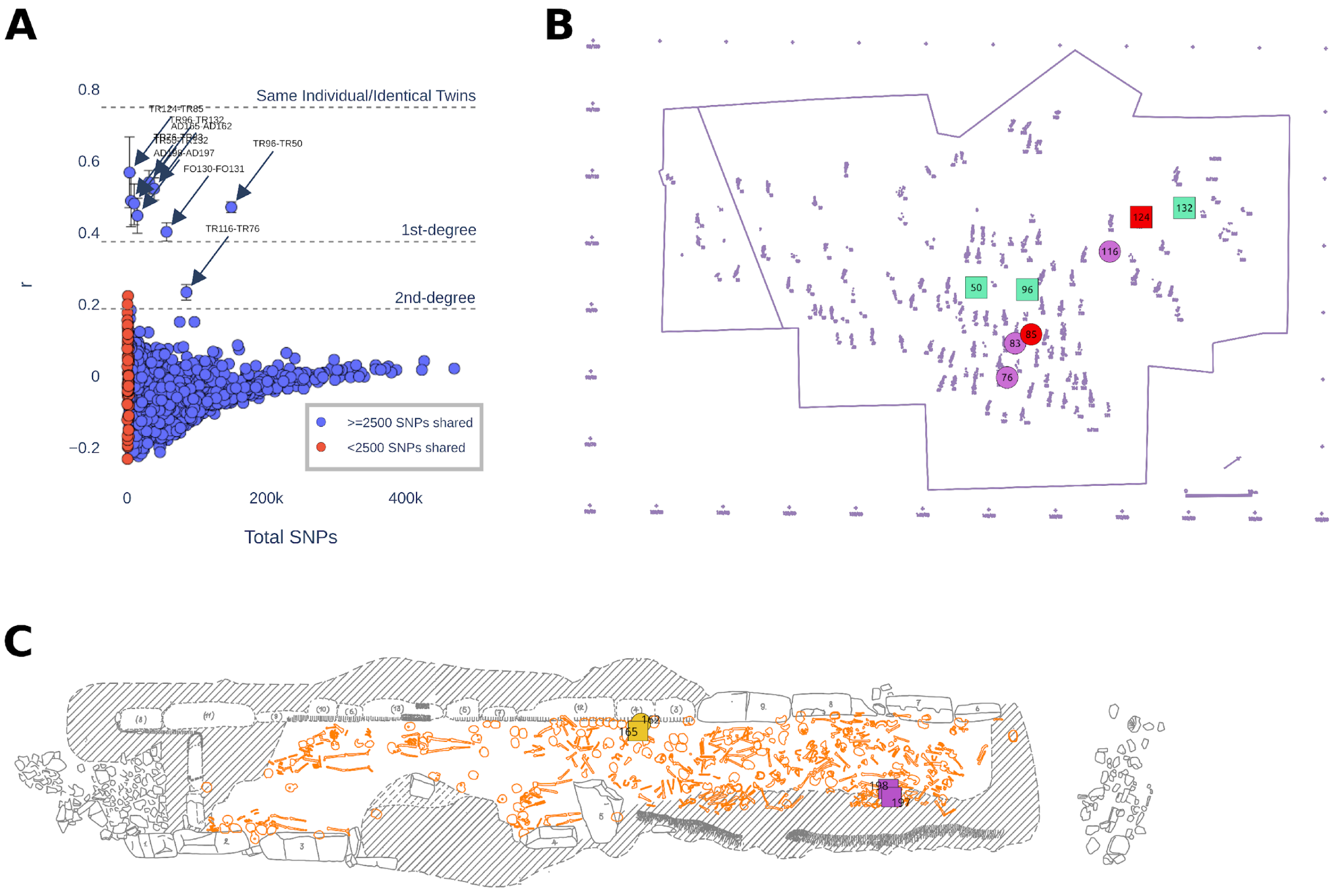

Fig S7. Results of kinship analysis. The first panel **(A)** displays the estimated relatedness coefficient (r) based on pairwise mismatch rates. Each marker represents one pairwise comparison. The x-axis shows the number of overlapping SNPs between each pair. The arrows point to the comparisons that correspond to the observed 1st and 2nd degree relationships described in the Results. Grave plan of the sites of Trebur **(B)** and Altendorf **(C)** are shown, with related individuals highlighted in the same color.

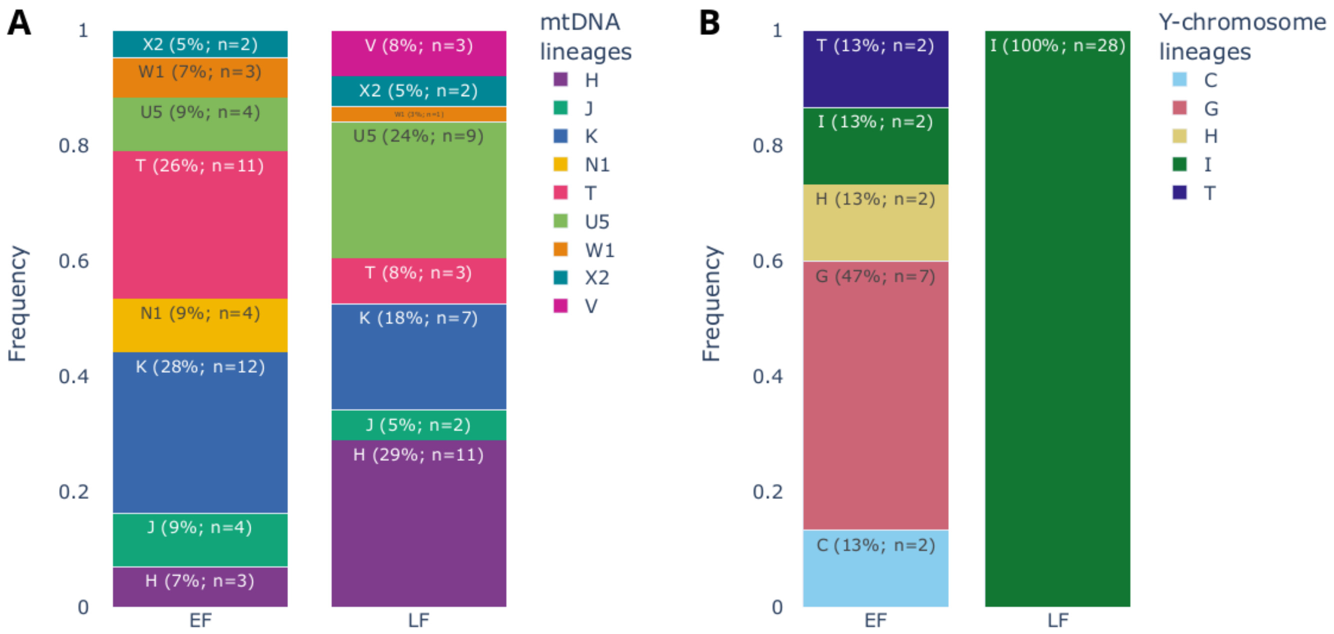

Fig S8. Maternal and paternal lineages. Frequency distribution of mtDNA **(A)** and Y-chromosome **(B)** macro-lineages in the early (EF) and late farmers (LF).

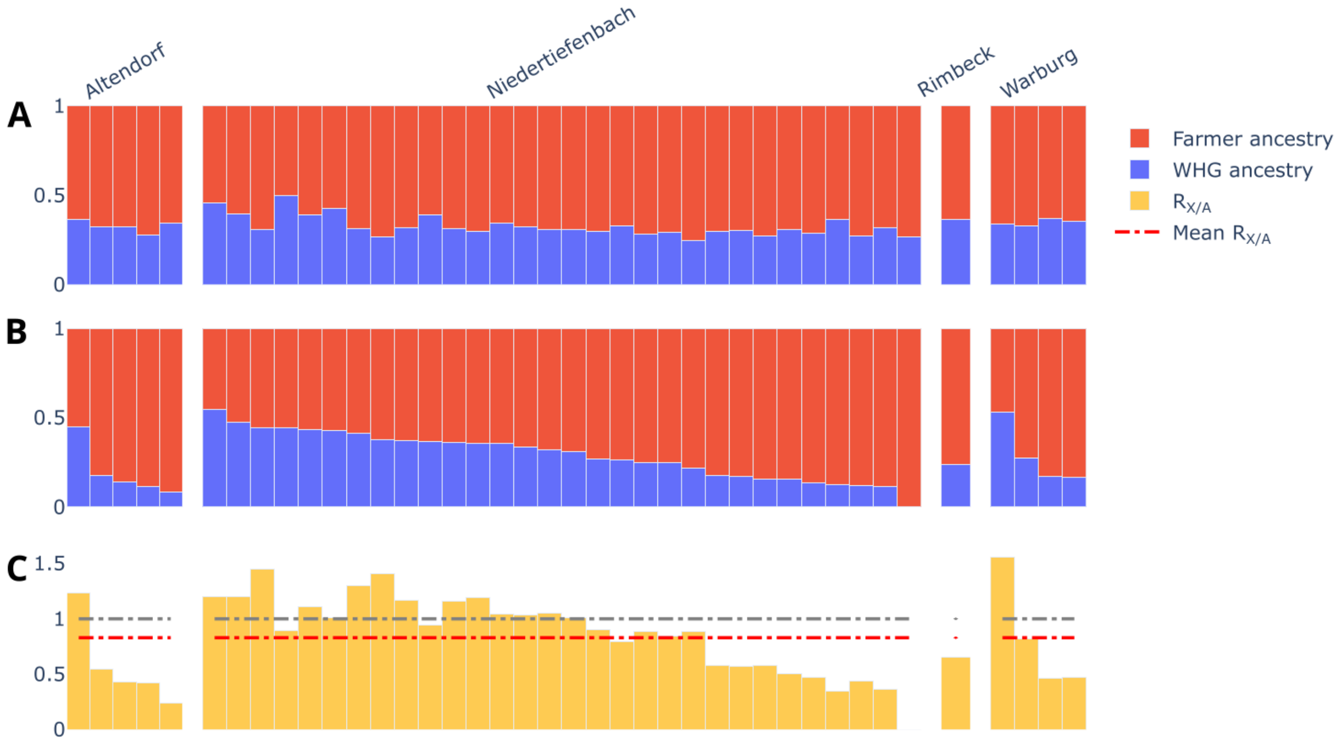

Fig S9. Results of the sex-biased admixture analysis in late farmers. Supervised clustering with Anatolian farmers and western hunter-gatherers (WHG) as sources: **(A)** using the mean proportions from 1000 resamples with 5388 autosomal SNPs and **(B)** using 5388 SNPs on the X-chromosome. **(C)** The ratio of X-chromosome to autosomal WHG ancestry ($R_{X/A}$) per sample. Each bar corresponds to one individual. The grey dashed line refers to the expected value of 1 for an equal contribution of male and female ancestors.

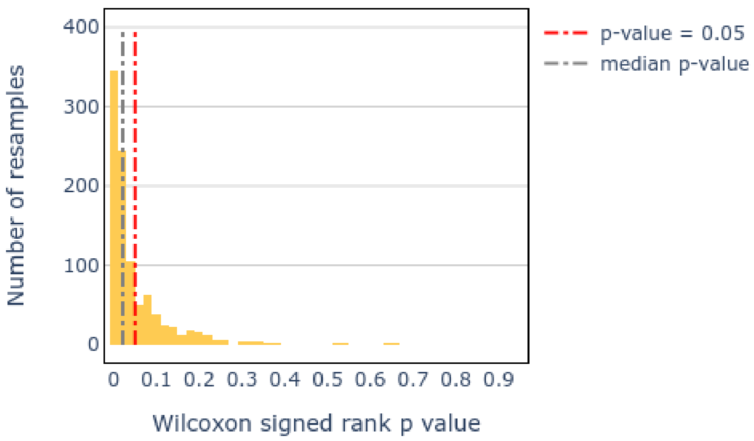

Fig S10. Statistical test results for the sex-biased admixture analysis. Histogram of p-values for the Wilcoxon sign-rank test performed for the distribution of WHG ancestry on the X-chromosome compared to 1000 autosomal ancestry distributions in late farmers (LF).

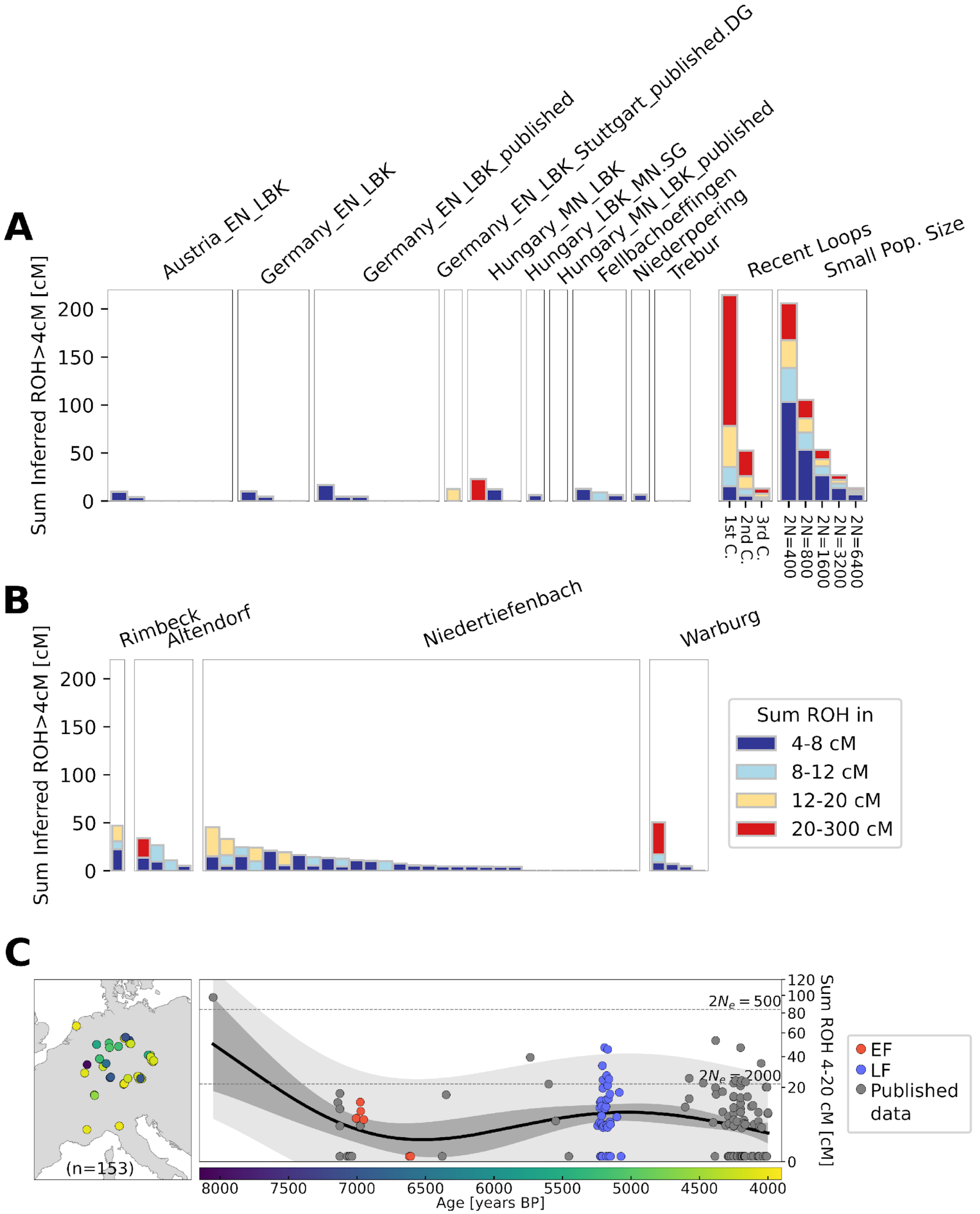

Fig S11. Estimation of runs of homozygosity (ROH). Stacked bar plots represent total length of ROH, stratified by different length categories in early farmers (EF) and published LBK sites in central Europe **(A)** and late farmers (LF) **(B)**. Expected ROH for offspring of parents being full cousins of degree 1, 2, 3 and for different effective populations sizes (2N) are also shown on the right side of panel A. **(C)** Sum of individual ROH (4-20cM) plotted along the timeline of the samples (EF in red, LF

in blue and reference published data in grey). The black solid line shows the average estimates, while the grey areas depict the 95% empirical confidence intervals for individuals (light grey) and estimated means (dark grey). Only samples with more than 400K covered SNPs were used in the ROH analysis.

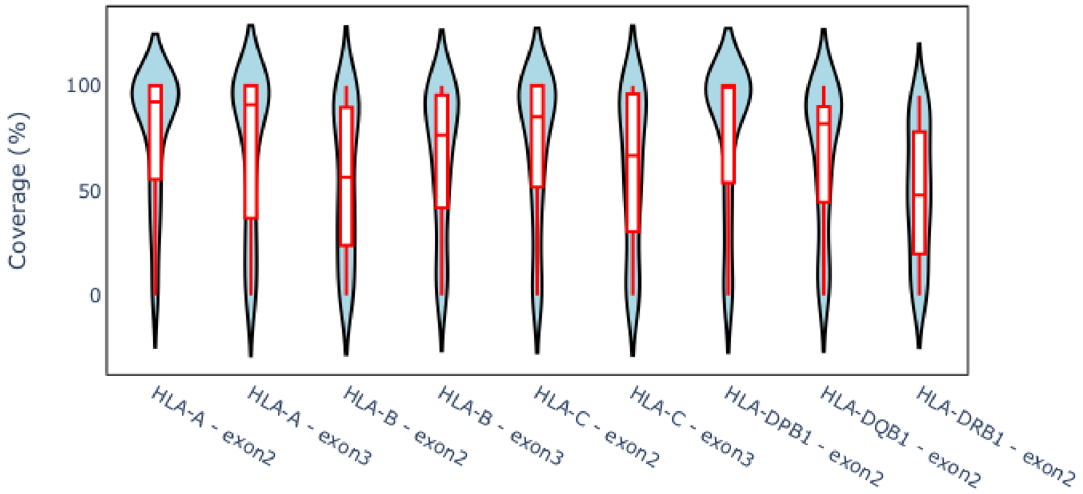

Fig S12. Coverage (%) for exons used for HLA typing. Violin and boxplots show breadth of coverage in the ancient samples analyzed in this study.

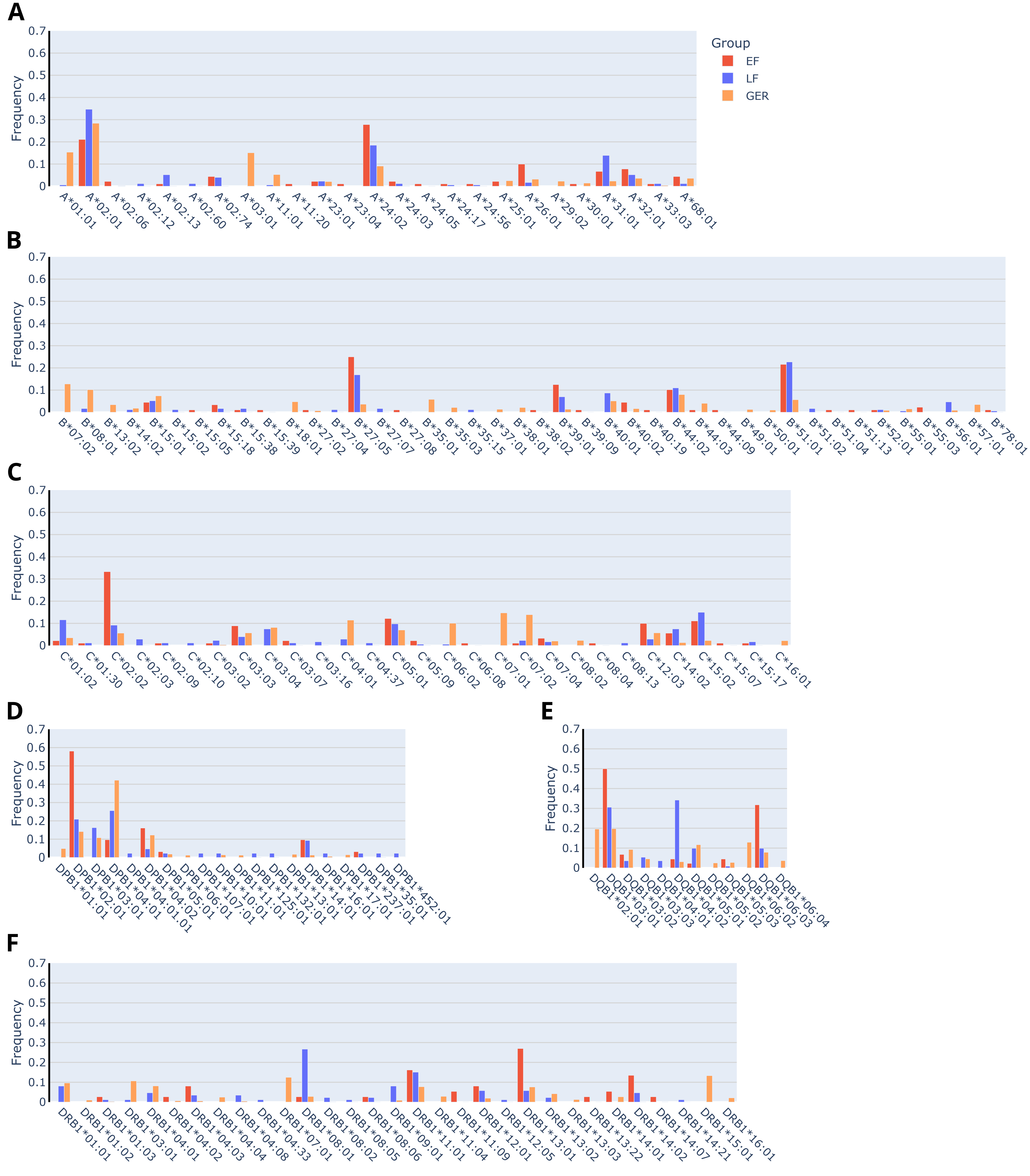

Fig S13. HLA allele frequencies. Frequency distribution of HLA-A (A), HLA-B (B), HLA-C (C), HLA-DPB1 (D) HLA-DQB1 (E), and HLA-DRB1 (F) alleles in early farmers (EF, red), late farmers (LF, blue) and modern Germans (GER, orange). Only alleles with a frequency above 1% in at least one of the groups are shown in panels A to F.

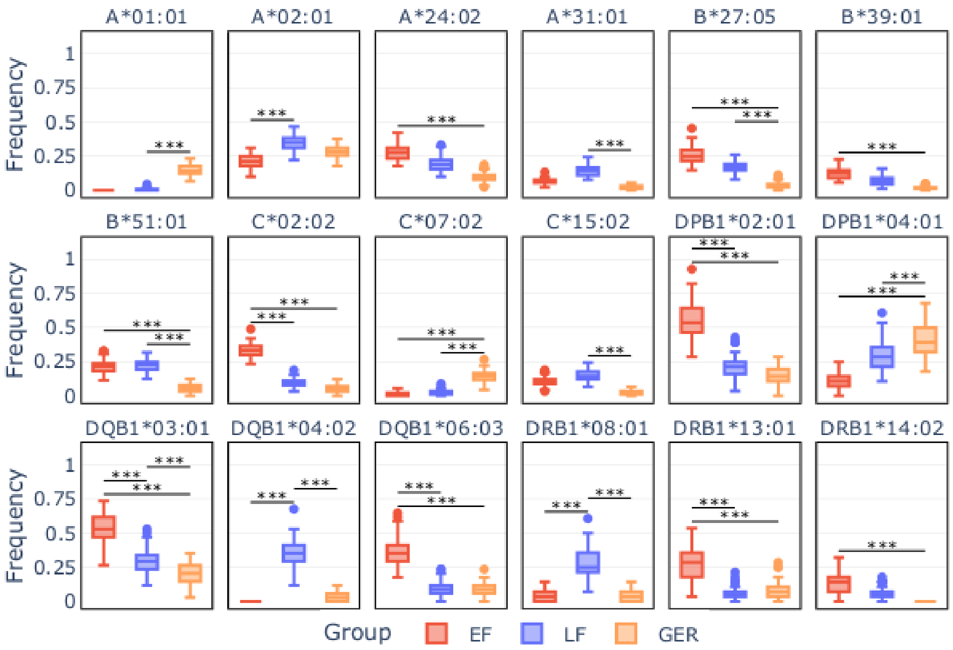

Fig S14. HLA alleles showing significant frequency differences after bootstrapping between early farmers (EF) and late farmers (LF) or between either Neolithic group and modern Germans. Frequency distribution of significantly different alleles across 100 resamples for EF, LF and GER. The Y-axis represents allele frequencies calculated separately for each HLA locus (i.e., HLA-A, -B, -C, -DPB1, -DQB1 and -DRB1). Significance was assessed using Mann-Whitney U test with multiple test correction (***: p≤0.001).

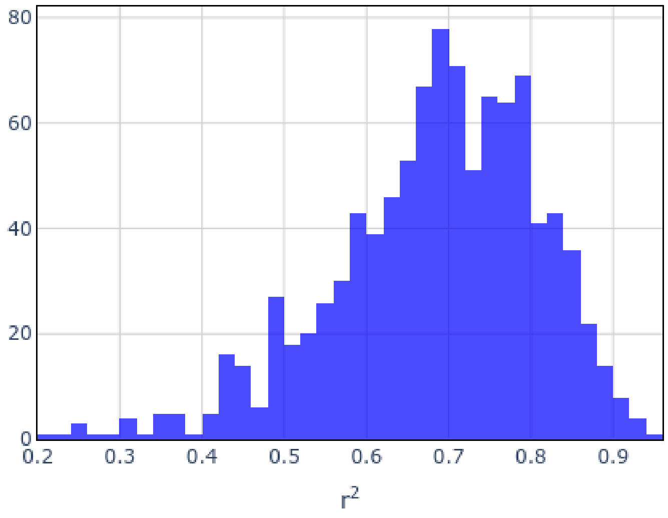

Fig S15. Robustness assessment of WHG ancestry estimation on the X-chromosome. Distribution of the r^2 statistic comparing X-chromosome ancestry estimates with those derived from 1,000 random down-sample iterations.

Table S1. List of identified related pairs in the newly reported sites.

Sample pair	Site	Shared SNPs	Mis-matches	Mis-match rate	r	r [95% CI lower bound]	r [95% CI upper bound]	Relatedness annotation
TR124-TR85	Trebur/ Hinkelstein	3523	654	0.186	0.568	0.470	0.667	1st-degree
TR96-TR50	Trebur/ Hinkelstein	149687	29668	0.198	0.471	0.456	0.486	1st-degree
TR96-TR132	Trebur/ Hinkelstein	31560	5971	0.189	0.541	0.508	0.574	1st-degree
TR116-TR76	Trebur/ Hinkelstein	85276	19531	0.229	0.234	0.212	0.255	2nd-degree
TR76-TR83	Trebur/ Hinkelstein	5685	1114	0.196	0.489	0.416	0.567	1st-degree
TR50-TR132	Trebur/ Hinkelstein	10280	2024	0.197	0.481	0.422	0.536	1st-degree
FO130-FO131	Fellbach Oeffingen	56662	11735	0.207	0.402	0.377	0.428	1st-degree
AD165-AD162	Altendorf	38855	7440	0.192	0.523	0.491	0.553	1st-degree
AD198-AD197	Altendorf	14875	2994	0.201	0.447	0.398	0.496	1st-degree

Table S2. Comparisons of Fst on the X-chromosome and autosomes to explore sex-biased admixture in late farmers (LF)

Comparison	Fst (autosomes)	Fst (chrX)	Q
LF-WHG	0.042	0.064	0.634
LF-Anatolia	0.025	0.033	0.755

Table S3. Genotype rate per HLA locus in early farmers (EF) and late farmers (LF)

Group	Locus	Genotype rate	# Alleles called
EF	HLA-A	1	90
EF	HLA-B	0.98	88
EF	HLA-C	1	90
EF	HLA-DPB1	0.38	34
EF	HLA-DQB1	0.44	40
EF	HLA-DRB1	0.44	40
LF	HLA-A	0.97	174
LF	HLA-B	0.96	172
LF	HLA-C	0.96	172
LF	HLA-DPB1	0.27	48
LF	HLA-DQB1	0.66	118
LF	HLA-DRB1	0.49	88

Table S4. HLA alleles with significant shifts in frequency. HLA alleles showing statistically significant shifts ($p \leq 0.05$, Fisher's exact test) and substantial frequency differences (≥10%) between early farmers (EF) and late farmers (LF), or between either Neolithic group and modern Germans (GER). Also shown are p-values from the Mann-Whitney U test based on 100 down-sampling iterations. In both tests, the p-values were corrected for multiple testing using the two-stage Benjamini and Hochberg step-up FDR-controlling procedure (TSBH-FDR).

HLA allele	Test	Frequency difference	p-value (Fisher's exact test)	p-value (Mann-Whitney U test)
A*02:01	EF vs LF	0.14	2.45e-02	2.60e-30
C*02:02	EF vs LF	0.24	7.40e-06	1.81e-33
DPB1*02:01	EF vs LF	0.37	1.98e-03	1.08e-32
DQB1*03:01	EF vs LF	0.19	2.78e-02	1.44e-29
DQB1*04:02	EF vs LF	0.3	1.05e-04	1.26e-37
DQB1*06:03	EF vs LF	0.22	2.01e-03	3.20e-33
DRB1*08:01	EF vs LF	0.24	1.57e-03	9.89e-33
DRB1*13:01	EF vs LF	0.21	2.58e-03	2.08e-28
A*24:02	EF vs GER	0.19	1.05e-06	1.86e-33
B*27:05	EF vs GER	0.21	5.55e-12	1.54e-33
B*39:01	EF vs GER	0.11	1.44e-07	9.83e-34
B*51:01	EF vs GER	0.16	1.40e-06	1.84e-33
C*02:02	EF vs GER	0.28	1.27e-14	1.81e-33
C*07:02	EF vs GER	0.13	7.37e-05	1.00e-33
DPB1*02:01	EF vs GER	0.44	6.48e-08	1.74e-33
DPB1*04:01	EF vs GER	0.33	2.27e-04	2.03e-33
DQB1*03:01	EF vs GER	0.3	1.99e-05	3.66e-33
DQB1*06:03	EF vs GER	0.24	1.24e-05	2.23e-33
DRB1*13:01	EF vs GER	0.19	4.88e-04	3.85e-24
DRB1*14:02	EF vs GER	0.13	6.56e-14	2.70e-37
A*01:01	LF vs GER	0.15	1.26e-10	2.14e-34
A*31:01	LF vs GER	0.12	3.06e-11	1.35e-33
B*27:05	LF vs GER	0.13	5.49e-11	1.84e-33
B*51:01	LF vs GER	0.17	1.01e-12	1.81e-33
C*07:02	LF vs GER	0.12	8.96e-07	3.12e-33
C*15:02	LF vs GER	0.13	4.80e-13	1.52e-33
DPB1*04:01	LF vs GER	0.17	3.02e-02	4.20e-12
DQB1*03:01	LF vs GER	0.11	7.07e-03	1.10e-13
DQB1*04:02	LF vs GER	0.31	2.06e-27	7.88e-34
DRB1*08:01	LF vs GER	0.24	4.60e-15	6.48e-33

Table S5. Most common HLA alleles (frequency ≥ 10%) in modern Germans (GER) compared to their frequencies in early (EF) and late farmers (LF).

HLA allele	Frequency in EF	Frequency in LF	Frequency in GER
A*01:01	0	0.006	0.154
A*02:01	0.211	0.347	0.284
A*03:01	0	0	0.151
B*07:02	0	0	0.128
B*08:01	0	0.017	0.102
C*04:01	0	0.029	0.115
C*06:02	0	0.006	0.101
C*07:01	0	0	0.147
C*07:02	0.011	0.023	0.139
DPB1*02:01	0.581	0.209	0.142
DPB1*03:01	0	0.163	0.108
DPB1*04:01	0.097	0.256	0.422
DPB1*04:02	0.161	0.047	0.123
DQB1*02:01	0	0	0.196
DQB1*03:01	0.5	0.306	0.198
DQB1*05:01	0.023	0.099	0.117
DQB1*06:02	0	0	0.129
DRB1*03:01	0	0.012	0.107
DRB1*07:01	0	0	0.125
DRB1*15:01	0	0	0.133
