# Supplementary material for: Admixture as a source for HLA variation in Neolithic European farming communities
Source: Genome Biol. 2025 Feb 28;26:43. doi: 10.1186/s13059-025-03509-6 (PMC11869582; doi:10.1186/s13059-025-03509-6)
Supplement: Supplementary file 3 — Additional file 3. Review history [file 13059_2025_3509_MOESM3_ESM.docx]

**1^st^ round**

**Reviewer 1**

Da Silva and colleagues generated 83 ancient low coverage genomes (average coverage 0.06X) enriched for HLA regions, These samples belonged to seven archeological sites across Germany, spanning the Early and Late Neolithic periods. Their analysis revealed shifts in HLA allele frequencies between these periods, likely attributed to the admixture of Late Neolithic populations with local Western Hunter-Gatherer (WHG) groups. The authors compared the same frequencies with present-day European populations. While the study's subject matter and scope are intriguing, I believe significant revisions are necessary before considering it for publication. Below, I outline my primary concerns.

The primary concern regards the reliability of the results given the exceptionally low coverage of the samples analysed in this study, particularly in mapping to complex and repetitive regions such as the HLA. Although the authors performed an enrichment on the HLA region further clarification is needed regarding how this enrichment strategy operates with ancient DNA and highly degraded genomes. It would be valuable to ascertain if the authors can establish a correlation between genotyping accuracy and sample coverage or if there are existing publications that address this specific issue. I see from the Supplementary table Data8 many samples with only few reads on some alleles. On the same line it is essential to understand how damage and transversions are accounted for in the analysis. Can the observed frequencies withstand when considering only transversions?


Moreover, the computational pipeline employed in the study seems insufficiently documented, with numerous gaps that avoid replication and understanding. I strongly recommend publishing a GitHub repository containing folders for each analysis to facilitate replication. Below, I detail several areas of concern.

- A comprehensive master table detailing the samples used for each analysis, along with the number of variants employed, is essential for transparency and reproducibility. Additionally, outlining the selection criteria for samples from the AADR (the version of the dataset should be included) is crucial and should be included in the Materials and Methods section and as Supplementary Data.
- Many of the software tools used in the analysis lack version information, such as smartpca, ADMIXTOOLS, and EIGENSOFT. Specifying the versions used would enhance the reproducibility of the study. Furthermore, clarification is needed regarding which mapping algorithm was utilised - whether it was Bwa-mem or Bwa-aln.
-For PCA analysis, it's important to indicate the number of variants used and the precise number of samples included in the analysis.
-Global ancestry proportion estimates: it's unclear why unsupervised ADMIXTURE analysis was not conducted for K=2 and 3, and clarification on this decision is required. Additionally, the rationale behind performing both unsupervised and supervised analyses needs to be elucidated, especially since only proportions from the supervised modality are referenced from a certain point. Exploring the results of the two ADMIXTURE modalities and qpAdm, comparing them, and implementing a method for selecting only concordant estimations between the methods would be beneficial. Regarding ADMIXTOOLS, clarification is needed on the version used and whether the authors employed the classic framework qpWave + qpAdm and tested all possible combinations of two, three, and four sources on the set of target populations. This is important to ascertain whether all models from the qpAdm analysis are supported and statistically significant Details regarding the specific options used should also be clarified and incorporated into the manuscript.
-Kinship analysis: Could the authors specify the number of bootstrap samples used to infer the 95% confidence interval (CI)?
-In the estimation of Shannon's diversity index, the authors need to clarify how they treated the EF, as the sample size should have remained consistent throughout the analysis.


Another significant aspect involves discussing the study results in the context of recent key publications in the field of HLA and archaeogenomics, such as Kerner et al., 2023, in Cell Genomics, which investigated signals of selection in genomic regions under pathogen-driven selection, and Barrie et al., 2024, in Nature, where local ancestry measures were estimated, including in HLA regions. The latter paper provided insights into the probability of regions, at the single base level, being introgressed from specific ancient ancestries, such as WHG. It would be intriguing to determine whether this applies to the specific alleles observed by the authors, either by employing the same framework used in these studies or by analyzing their summary statistics for the relevant alleles.

The manuscript lacks essential archaeological context for the samples, failing to provide explanations of the extraction sites and their cultural backgrounds. While Supplementary Data 1 offers valuable insights, it should be more extensively discussed in the main text, especially regarding groups like the Middle Neolithic Hinkelstein and Großgartach, and their relevance to the reported samples. As the authors introduce 83 ancient genomes for the first time, it's imperative to include pertinent contextual information. Furthermore, details about previously published Niedertiefenbach individuals should be integrated into this section, rather than presenting it as a separated paragraph.

In general, the results section appears to be structured more like a list of applied methods, lacking a cohesive narrative based on the obtained results. It would be beneficial to reframe this section to provide a coherent and cohesive story - with paragraph titles - that accurately reflects the findings.

Minor points:
Line 29-30: I suggest using "local" western hunter-gatherers (WHG)
Line 32: add "archeological" to EF LF sites
Line 44: : I suggest to cite Marchi et al., 2022 Cell, and include the publication dates for completeness.
Line 63-65: Place the study in the broader context of Allentoft et al., 2024, Nature, and Barrie et al., 2024, Nature.
Line 69. Ensure consistency in reporting the number of individuals throughout the manuscript. Mention that although sequences were generated for 125 individuals, only 83 passed quality control (QC), and 75 were enriched for the HLA region. Moreover, clarify the criteria for selection on the 75 individuals.
Line 313: Specify from which bones and/or teeth the samples have been extracted and ensure that Supplementary Data 1 is cited along the text.
Line 78. Include information about the Niedertiefenbach samples in this section, as they have been published and analyzed.
Line 87: Clarify, here too, which genome reference was used for aligning the genomes.
Line 88. Clarify whether "Datasets" or "samples" are being referred to.
Line 90: If PCA projection was performed, refer to it in this section and add a reference to the Materials and Methods section.
Line 142: Comment more extensively on the inferred haplogroups, especially in relation to other phylogenetic studies from the same region, such as Olalde et al., 2015, MBE.
Q statistic function and its elements require clearer explanation in the Materials and Methods section.
Clarification is needed regarding why ROH was inferred for only 39 LF individuals.
Line 164: LBK individuals were included previously. Did the authors mean LBK from other regions?
Line 175: Consider using acronyms like "NT" for Niedertiefenbach, when already defined.
Line 186: To enhance the evidence supporting the claim of changes in HLA alleles after WHG admixture, it's suggested to provide a better overview of these changes, possibly by including more panels in Figure 3. Specifically, the author should consider moving some figures from the supplementary material to the main text.
A citation is needed for the statement "However, the hypothesis remains to be further tested once true HLA calls become available for WHG." Clarify what the authors mean.
At Line 262, the authors should specify which haplotype they are referring to.
On Line 309, the number on the table is 152.
There seems to be confusion regarding the proportions in LF, as stated: "LF, who are represented by 110 WBC in this study," which contradicts the presence of other LF individuals. This discrepancy needs clarification.

**Reviewer 2**

The paper reports the population genomic structure of the early farmers (EF) from Anatolia (7500 BP) and the late farmers (LF) formed by the admixture of EF and western hunter-gatherers (WHG). Notably, the effect of WHG's admixture on LF's HLA repertoire has not been studied before, and the article focuses on the differences in HLA profiles of EF and LF, compares them with modern Europeans, accurately depicts the immune characteristics of the ancients, and demonstrates correlation with the admixture of WHG and the virus selection pressure. The added value to this paper is the newly generated genome-wide data of 83 individuals from six archaeological sites covering the 2300-year transition from the Early (LBK) to the Late Neolithic, which provides a high-quality data reference for the study of ancient DNA in the Neolithic age. However, several concerns are to be considered and addressed.

Major:
1. This study has interesting observation on the increased HLA diversity from early farmer to late farmers by generating and analyzing new HLA variation data of 79 individuals. The authors correlate such diversity increase to WHG admixture, though the correlation between HLA diversity and WHG admixture does not necessarily mean causal. It would be nice to see more solid evidence in the correlation or causal test.

2. The authors also reported that both EF and LF exhibited a relatively narrow HLA allele spectrum compared to today, which in authors' opinion coincides with sparse traces of pathogen DNA, potentially indicating a lower pathogen pressure at the time. It is worth discussing whether it is the fact of sparse traces of pathogen DNA or just not big enough dataset or sampling bias (in the later two cases we can rarely find pathogen DNA)..

3. The authors also found drastic changes in Y-haplogroups from EF to LF and sex-biased admixture in LF. Regarding the sex bias, has the authors tested with other approaches, apart from Rx/A ratio. I wonder how would the data quality (e.g. X-chromosome coverage) affects the robustness of estimates?

4. In the analysis of ROH fragments and effective population size, EF has a shorter total length of ROH and a larger effective population size than LF, and according to the change of haplotypes ratio on the sex chromosome, so the author believed that there was consanguineous marriage in LBK (that is, the period from EF to LF). Counting the difference between EF and LF, the author proposed a hypothesis that EF itself had the custom of consanguineous marriage, which led to the decrease of effective population and the accumulation of Roh fragment length until the LF formed as a result of mixing with WHG , which failed to improve this situation significantly. Given this problem, I think there are two aspects to be analyzed: First, we can view the existing EF data to be subdivided in time scale, and check whether the late EF population has a smaller effective population size or a longer Roh segment than the earlier EF population (regardless of environmental changes), which, to some extent, can support the hypothesis of consanguineous marriage in LBK period; Second, we can check the Y chromosome typing status of WHG to answer the question why with a large number of WHG mixed in, especially when the author also made sex-bias analysis at the same time and believed that the large number of WHG mixed in favor of men, there is still only one y haplotypes?

5. The author believes that the frequency change of LF compared with EF in the HLA region is mainly from the mixing of WHG, and the basis is that, in line 253, DQB1 locus(within HLA-II) is enriched in the individuals with WHG ancestors in the Neolithic age, while the major frequency 252 changes (>20%) between EF and LF mainly affect HLA class II alleles. Through the previous analysis, we can see that there are some WHG individuals in this study (loschbour Luxembourg, Bichon Switzerland, and one individual from Mont aim é/paris102 basin), I think these individuals can be directly used together with EF to calculate the introgression fragments of the two in LF, which can more directly explain the causal relationship.

6. The typing of HLA uses a combination of OptiType and TARGT pipeline. Although TARGT is a method for ancient and modern shotgun sequence data with moderate to low sequence coverage, OptiType is not. Why is it combined with OptiType, or why is it not combined with more HLA typing methods?

7. The preservation condition of ancient DNA may have potential impacts on the results. Sensitivity analysis can be performed on samples with different preservation conditions to assess their impact on HLA gene typing and diversity estimation.

8. Only alleles consistently called by both methods were included to ensure a higher reliability of the results. However, OptiType can only type class I genes(HLA-A, -B and -C). How are the results for class II (HLA-DPB1, -DQB1 and -DRB1) handled? Are there any statistics on the consistency of typing between OptiType and TARGT? If it's not very high, it may indicate that even the loci with consistent typing are not very reliable. It is suggested to check the read coverage and depth at several loci for enhanced reliability.

9. HLA polymorphism increases significantly with sample size. For the alleles with significant frequency differences found in EF, LF, and GER, how to exclude the influence of the number of samples and sampling bias? Multiple random downsamplings could be conducted to determine if the results remain consistent.

10. It is advisable to consider and discuss other factors that may influence HLA diversity, such as environmental pressure, in addition to admixture.

minor:
11. To solve the problem that the mixed model of WHG estimated the mixing time with dates is not accurate, MultiwaverX software can be used, which supports the construction of a gender-biased mixed model and the construction of a mixed model with continuous mixed gene flow.

12. As for the characterization of the diversity of different groups in the HLA region, we can also try to use nucleotide diversity and haplotype diversity.

**Authors’ response**

We cordially thank the reviewers for their time and insightful comments, which have significantly improved our manuscript entitled "Admixture as a source for HLA variation in Neolithic European farming communities".

We have meticulously revised the manuscript, restructuring it according to the reviewers' recommendations. Additionally, we have expanded the Results, Methods and Discussion sections. We greatly value the reviewers' input and have responded to all their comments.

Below, we address each comment separately (responses in green), specifying the revisions made and indicating where the changes can be found in the main text (line number). For clarity, we have split some of the more extensive comments into smaller sections to address each point separately. Additionally, we have numbered Reviewer #1’s comments to facilitate easy reference in other responses when necessary. The full references for the cited papers are provided at the end of the letter.

**Reviewer reports:**

**Reviewer #1:** Da Silva and colleagues generated 83 ancient low coverage genomes (average coverage 0.06X) enriched for HLA regions, These samples belonged to seven archeological sites across Germany, spanning the Early and Late Neolithic periods. Their analysis revealed shifts in HLA allele frequencies between these periods, likely attributed to the admixture of Late Neolithic populations with local Western Hunter-Gatherer (WHG) groups. The authors compared the same frequencies with present-day European populations. While the study's subject matter and scope are intriguing, I believe significant revisions are necessary before considering it for publication. Below, I outline my primary concerns.

**1.** The primary concern regards the reliability of the results given the exceptionally low coverage of the samples analysed in this study, particularly in mapping to complex and repetitive regions such as the HLA. Although the authors performed an enrichment on the HLA region further clarification is needed regarding how this enrichment strategy operates with ancient DNA and highly degraded genomes. It would be valuable to ascertain if the authors can establish a correlation between genotyping accuracy and sample coverage or if there are existing publications that address this specific issue. I see from the Supplementary table Data8 many samples with only few reads on some alleles. On the same line it is essential to understand how damage and transversions are accounted for in the analysis. Can the observed frequencies withstand when considering only transversions?

Our genotyping method is based on the combination of the TARGT pipeline and OptiType. Both tools have been used in ancient DNA studies before successfully. The TARGT pipeline was specifically developed to analyze low-coverage shotgun sequence data. The performance of the HLA enrichment strategy as well as the correlation between coverage, read depth and genotyping accuracy have been analyzed in a proof-of-concept article using both ancient samples and simulations (please see Figures 2, 4, S1-S5 in Pierini et. al., 2020). The analysis in that paper showed that while it is true that increased coverage leads to higher genotype success and accuracy, even a few reads are sufficient to yield reliable allele calls with the TARGT pipeline. This is mainly achieved by the manual curation of the mapped reads, allowing inspection of the alignments. Furthermore, this manual inspection step allows the identification of ancient DNA damage that is usually enriched towards the ends of reads. We also trim the terminal two bases from reads to control the impact of aDNA damage on allele calls. In addition, a recent benchmarking study revealed that OptiType performs better with low-coverage data compared to other fully automated algorithms (Thuesen et. al., 2022).

Although using only transversions is a common practice in SNP-based aDNA analysis, it would be counterproductive to apply it to our approach. Excluding transitions would make it impossible to distinguish some HLA alleles, especially at resolutions higher than 1-field. Also, many polymorphic positions across the HLA genes do not fit into the classical biallelic categorization as more than two nucleotides can be observed in one position. Therefore, excluding transitions would result in a bias toward calling alleles that are differentiated mainly by transversions.

In the Methods section, we have modified and expanded the paragraph on HLA genotyping, highlighting the reliability of both TARGT and OptiTpye for HLA typing with ancient DNA (lines 551-562).

**2.** Moreover, the computational pipeline employed in the study seems insufficiently documented, with numerous gaps that avoid replication and understanding. I strongly recommend publishing a GitHub repository containing folders for each analysis to facilitate replication. Below, I detail several areas of concern.

We agree that more details are necessary to allow proper replication. We have made the following changes to the Methods section:

Line 411-412: added information on filter used for mtDNA determination.

Lines 416-419: clarified that two datasets were generated (one with the Human Origins panel and the other with the 1240K panel) and which analysis used what.

Lines 420-422: expanded the information on PCA (ie: # SNPs used, # of individuals)

Line 429-430: details added to the filter used in the outgroup f3 statistics.

Line 432-466: more details were provided for several admixture analyses performed (please also see comment **#6.1** and **#6.2** below).

Line 469-476: more information about the statistic Q was provided.

Line 524-549: added new section detailing imputation and chromosome painting analysis (see comment **#9.2** below).

Line 551-562: Added more details on the HLA genotyping.

Line 583-588: Added paragraph detailing how we associated certain alleles with WHG ancestry (see comment **#9.2** below).

Line 599-604: Clarified how the resampling for the Shannon index analysis was done.

In addition, we are happy to provide the necessary files and scripts for each analysis upon request. For this purpose, we have added the following statements in line 630:

"Scripts and files used for each analysis are available upon request. Please send the request to the corresponding author at [b.krause-kyora@ikmb.uni-kiel.de](mailto:b.krause-kyora@ikmb.uni-kiel.de)"

**3.** - A comprehensive master table detailing the samples used for each analysis, along with the number of variants employed, is essential for transparency and reproducibility. Additionally, outlining the selection criteria for samples from the AADR (the version of the dataset should be included) is crucial and should be included in the Materials and Methods section and as Supplementary Data.

To increase transparency and improve reproducibility, we have added more tables listing the samples and populations used for each analysis separately:

Sup Data 11: list of individuals used for imputation and chromosome painting

Sup Data 13: list of samples used for PCA

Sup Data 14: list of populations used for outgroup f3 statistics

Sup Data 15: list of populations used for the unsupervised ADMIXTURE

Sup Data 16: list of populations used for supervised ADMIXTURE

Additionally, we have expanded the Methods section by adding more details on the selection criteria for using the AADR groups (please see lines referenced in the previous comment **#2**, especially regarding the PCA and admixture analyses).

We would also like to point out that we used the version 50.0.p1 of the AADR (line 417).

**4.** - Many of the software tools used in the analysis lack version information, such as smartpca, ADMIXTOOLS, and EIGENSOFT. Specifying the versions used would enhance the reproducibility of the study. Furthermore, clarification is needed regarding which mapping algorithm was utilised - whether it was Bwa-mem or Bwa-aln.

The missing version information of the following tools has been included in the Methods section:

Line 410: HaploGrep2 v2.4.0

Line 411: yHaplo v1.1.2.

Line 420: smartpca v16000

Line 420: EIGENSOFT v7.2.1

Line 426: qp3Pop v650

Line 427: Admixtools v7.0.1

Line 442: qpWave v1200

Line 442: qpAdm v1201

Line 453: DATES v753

Line 513: HapROH v0.3a4

Line 521: skbio v0.5.6 (Python module)

Line 552: TARGT v2

Line 553: OptiType v1.2.1

Line 590: vegan v2.6.4 (R package)

Additionally, we used BWA-ALN for the alignment (line 386).

**5.** -For PCA analysis, it's important to indicate the number of variants used and the precise number of samples included in the analysis.

We have added the requested information (597,573 SNPs and 1154 individuals) to lines 420-422 of the Methods section.

**6.1.** -Global ancestry proportion estimates: it's unclear why unsupervised ADMIXTURE analysis was not conducted for K=2 and 3, and clarification on this decision is required.

The unsupervised admixture was initially performed using K=3 through 12. Supplementary Figure 3 only displayed the Ks with the lowest cross-validation errors (CVE; K=4 - 7). We have originally not included K=2 as it yields high CVE and it is not very informative. However, we have now included unsupervised admixture with K=2 to our analysis and modified Supplementary Figure 3 to show all calculated Ks (2-12) (also shown in **Figure 1** below). Additionally, we provide a separate plot with the CVE for each K (new Supplementary Figure 4 and **Figure 2** below).

**Figure 1. Results from unsupervised admixture.** The analysis was performed using 2-12 components (K) for 295 selected ancient and modern populations/individuals (only 11 are plotted). Each K was run with 100 bootstraps. HG = Hunter-gatherer; N = Neolithic; EN = Early Neolithic; MN = Middle Neolithic; EBA = Early Bronze Age; LBK = Linear Pottery culture.

**Figure 2. Cross-validation errors for each K modelled with unsupervised admixture.**

**6.2.** Additionally, the rationale behind performing both unsupervised and supervised analyses needs to be elucidated, especially since only proportions from the supervised modality are referenced from a certain point. Exploring the results of the two ADMIXTURE modalities and qpAdm, comparing them, and implementing a method for selecting only concordant estimations between the methods would be beneficial. Regarding ADMIXTOOLS, clarification is needed on the version used and whether the authors employed the classic framework qpWave + qpAdm and tested all possible combinations of two, three, and four sources on the set of target populations. This is important to ascertain whether all models from the qpAdm analysis are supported and statistically significant Details regarding the specific options used should also be clarified and incorporated into the manuscript.

The unsupervised ADMIXTURE analysis was intended to be used as a starting point to explore population structure in a hypothesis-free manner, testing models with several components (K). Based on the unsupervised ADMIXTURE results, which showed that our studied populations carry components that are maximised in Anatolian farmers and WHG proxies (see models with lowest CVE: K=5 and K=6), we applied a qpWave model using two sources (Turkey_N and Luxembourg_Loschbour) plus our six groups as left populations. The qpWave results confirmed that 2 streams of ancestries were sufficient to fit our data well (rank1 p-value>0.05 for all our EF and LF groups; Supplementary Data 3). We then proceeded to model their ancestry proportions with qpAdm in a hypothesis-driven manner, choosing to test specific models that were considered most archaeologically plausible/relevant for our set of target populations.

Before, we provided the qpAdm results only for the models using Luxembourg Loschbour as WHG proxy. We have now changed that and added all model combinations results tested to Supplementary Data 4. Rather than mentioning estimates from any specific model, we have now calculated the average farmer and WHG contributions across all feasible two-way models and have consistently used these averages instead throughout the text (unless stated otherwise). We have also updated Supplementary Figure 5 (**Figure 3** below) with these estimates.

We have expanded both the Results and Methods, providing more details as described above (lines 111-116, 432-466).

We would also like to point out that specifically for Figure 1C and the analysis of sex-biased admixture, we utilised supervised ADMIXTURE models for ancestry estimation on an

individual sample basis rather than qpAdm (following Goldberg et. al., 2017). We have added this information in the Methods section (lines 459-466, 485-486).

The version of ADMIXTOOLS as well as more details on the options used were included as follows:

Line 427: Admixtools v7.0.1

Line 442: qpWave v1200

Line 442: qpAdm v1201

Line 447-448: “The qpAdm program was run with the following options: “allsnps: NO”.”

**Figure 3. Two-way qpAdm-based admixture models displaying western hunter- gatherer (WHG) and farmer ancestries**. The ancestry estimates are based on the average of feasible models using several WHG and farmer proxies (**Supplementary Data 4**). The error bars show the standard errors across the used models.

**7.** -Kinship analysis: Could the authors specify the number of bootstrap samples used to infer the 95% confidence interval (CI)?

We have added this information (1000 bootstraps) to line 503.

**8.** -In the estimation of Shannon's diversity index, the authors need to clarify how they treated the EF, as the sample size should have remained consistent throughout the analysis.

This is correct. For the EF, rather than the downsampling that was done for the other groups, a resampling (with replacement) was performed for them. This information was added to lines 599-604.

**9.1.** Another significant aspect involves discussing the study results in the context of recent key publications in the field of HLA and archaeogenomics, such as Kerner et al., 2023, in Cell Genomics, which investigated signals of selection in genomic regions under pathogen-driven selection, and Barrie et al., 2024, in Nature, where local ancestry measures were estimated, including in HLA regions.

In the Discussion section, we have referred to both suggested papers in the context of our results in lines 328-330 and 352-354.

**9.2.** The latter paper provided insights into the probability of regions, at the single base level, being introgressed from specific ancient ancestries, such as WHG. It would be intriguing to determine whether this applies to the specific alleles observed by the authors, either by

employing the same framework used in these studies or by analyzing their summary statistics for the relevant alleles.

We have now performed local ancestry inference analysis in imputed and well covered LF individuals (details added to Methods lines 524-549). We observed a substantial excess of WHG ancestry within the larger MHC region (chr6: 28477797-33448354) (**Figure 4**), supporting our hypothesis and corroborating previous studies (Barrie et. al., 2024, Davy et. al., 2023).

**Figure 4**. Local estimate of western hunter-gatherer (WHG) ancestry within the MHC region (highlighted in green) and the surrounding 5-Mb flanking regions on either side. The grey dashed line represents the mean genome-wide WHG ancestry.

Furthermore, we investigated the local ancestry of each individual across the MHC region in relation to the three alleles (A*02:01, DQB1*04:01 and DRB1*08:01) that increased in frequency from EF to LF. We hypothesized that this increase was the result of introgression from WHG. In support of this hypothesis we found that individuals with DQB1*04:01 and DRB1*08:01 alleles, which form a tightly linked haplotype, have a much higher probability of carrying WHG ancestry across the MHC region (**Figure 5**).

**Figure 5.** Comparison of the mean WHG ancestry across the MHC region between carriers (denoted by +) and non-carriers (denoted by -) of alleles A*02:01, DRB1*08:01 and DQB1*04:01. Bonferroni-corrected p-values of the Mann-Whitney test are indicated above boxplots.

These new findings were added to the Results section (lines 214-223).

**10.** The manuscript lacks essential archaeological context for the samples, failing to provide explanations of the extraction sites and their cultural backgrounds. While Supplementary Data 1 offers valuable insights, it should be more extensively discussed in the main text, especially regarding groups like the Middle Neolithic Hinkelstein and Großgartach, and their relevance to the reported samples. As the authors introduce 83 ancient genomes for the first time, it's imperative to include pertinent contextual information. Furthermore, details about previously published Niedertiefenbach individuals should be integrated into this section, rather than presenting it as a separated paragraph.

We have now added relevant information about the archaeological context for the six sites for which we reported new data in the Supplementary Information document (Supplementary Note 1). As we provide more extensive archaeological information than the main text could accommodate, it was included in the supplement instead. We now cite the Supplementary Note 1 in the Results and Methods section of the main text in line 85.

**11.** In general, the results section appears to be structured more like a list of applied methods, lacking a cohesive narrative based on the obtained results. It would be beneficial to reframe this section to provide a coherent and cohesive story - with paragraph titles - that accurately reflects the findings.

We have followed your suggestions and rephrased the section headers to reflect the findings (lines 89, 107, 133, 142, 154, 163, 182, 194, 214, 225, 237). By doing so, we have improved the text’s cohesion.

Minor points:

**12.** Line 29-30: I suggest using "local" western hunter-gatherers (WHG)

This suggestion was incorporated in line 29.

**13.** Line 32: add "archeological" to EF LF sites

This suggestion was incorporated in line 32.

**14.** Line 44: I suggest to cite Marchi et al., 2022 Cell, and include the publication dates for completeness.

We have included this reference to line 47.

**15.** Line 63-65: Place the study in the broader context of Allentoft et al., 2024, Nature, and Barrie et al., 2024, Nature.

We have extended the second paragraph in the Introduction to include Barrie et. al., 2024, Irving-Pease et. al., 2024 as well as Davy et. al., 2023 (line 66). We discuss our findings in the context of these publications in the Discussion (lines 304, 352). We would like to mention that Barrie et al. and the companion papers Allentoft et. al., 2024 / Irving-Pease et. al., 2024 had not been published when we submitted our manuscript.

**16.** Line 69. Ensure consistency in reporting the number of individuals throughout the manuscript. Mention that although sequences were generated for 125 individuals, only 83 passed quality control (QC), and 75 were enriched for the HLA region. Moreover, clarify the criteria for selection on the 75 individuals.

For consistency, we have now reported the true sample size (i.e., samples that passed QC) throughout the text: 83 for population genetic analyses (>20,000 SNPs from 1240K panel) and 79 for HLA frequency/diversity analyses (individuals for whom a genotyping call was possible).

We clarify that 95 individuals were enriched (HLA-capture) and 79 of these were successfully genotyped for HLA (line 195-198).

**17.** Line 313: Specify from which bones and/or teeth the samples have been extracted and ensure that Supplementary Data 1 is cited along the text.

We have done as suggested and cited the Supplementary Data 1 more often along the text (lines 76, 85 ,93, 136, 159, 202, 240, 275, 365, 375, 570).

**18.** Line 78. Include information about the Niedertiefenbach samples in this section, as they have been published and analyzed.

To prevent confusion of the readers, we included information about the Niedertiefenbach samples later in the Results section (line 142-152). This approach was deemed necessary because we first conducted population genetic analysis exclusively on the newly generated samples from this study, and only afterward did we incorporate the Niedertiefenbach samples into the LF group for the remaining analyses (e.g., sex-biased admixture, ROH, HLA analyses).

**19.** Line 87: Clarify, here too, which genome reference was used for aligning the genomes.

This information is already available in the Methods section (i.e., build hg19; line 385). We adhered to your suggestion and also mentioned it in the Results section in line 90.

**20.** Line 88. Clarify whether "Datasets" or "samples" are being referred to.

We have changed “datasets” to “samples” in line 91.

**21.** Line 90: If PCA projection was performed, refer to it in this section and add a reference to the Materials and Methods section.

We have added the information that the ancient samples are projected onto the modern West-Eurasian variation in lines 93-94.

**22.** Line 142: Comment more extensively on the inferred haplogroups, especially in relation to other phylogenetic studies from the same region, such as Olalde et al., 2015, MBE.

We have now added a separate subsection in the Results part describing the changes in the mtDNA and Y chromosome haplogroup compositions that occurred from EF to LF (lines 154-161). Additionally, we have added a paragraph to the Discussion (lines 273-281).

**23.** Q statistic function and its elements require clearer explanation in the Materials and Methods section.

More details on the Q statistic were added to the Methods section (lines 469-476).

**24.** Clarification is needed regarding why ROH was inferred for only 39 LF individuals.

We chose not to add other public Late Neolithic individuals to the LF group for this analysis because we wanted to focus on WBC-associated individuals. We added this rationale to the Methods line 517-518.

**25.** Line 164: LBK individuals were included previously. Did the authors mean LBK from other regions?

Yes. We have included data from published LBK sites (ROH results produced by Ringbauer et. al., 2021). We have added this reference to make it clearer in lines 186

.

**26.** Line 175: Consider using acronyms like "NT" for Niedertiefenbach, when already defined.

Although Niedertiefenbach is one of those terribly long German names, we would like to write it out to avoid an additional abbreviation. Additionally, we would need to abbreviate the other site names for consistency as well.

**27.** Line 186: To enhance the evidence supporting the claim of changes in HLA alleles after WHG admixture, it's suggested to provide a better overview of these changes, possibly by including more panels in Figure 3. Specifically, the author should consider moving some figures from the supplementary material to the main text.

We moved Supplementary Figures 14 to the main manuscript as a new Figure 3. Additionally, we included two new figures to the main text showing the local ancestry results (Figure 4 and 5).

**28.** A citation is needed for the statement "However, the hypothesis remains to be further tested once true HLA calls become available for WHG." Clarify what the authors mean.

We have deleted this sentence since we have now tested the hypothesis using local ancestry inference (see comment **#9.2**).

**29.** At Line 262, the authors should specify which haplotype they are referring to.

We believe the sentence is clear: the haplotype is formed by the two alleles mentioned in the sentence (lines 313-315):

“Interestingly, the alleles with high frequencies in EF, DRB1*13:01 (27%) and DQB1*06:03 (32%), whose co-occurrence indicates a haplotype, have been shown to be protective against viral hepatitis A (HAV) and B (HBV) infections today.”

**30.** On Line 309, the number on the table is 152.

We have corrected the number in the text (n=175). The number in the table is correct.

**31.** There seems to be confusion regarding the proportions in LF, as stated: "LF, who are represented by 110 WBC in this study," which contradicts the presence of other LF individuals. This discrepancy needs clarification.

We have added a header to this Results section clarifying that our group referred to as LF is exclusively composed of WBC individuals who present higher WHG ancestry than other contemporaneous groups (line 107).

**Reviewer #2:**

The paper reports the population genomic structure of the early farmers (EF) from Anatolia (7500 BP) and the late farmers (LF) formed by the admixture of EF and western hunter-gatherers (WHG). Notably, the effect of WHG's admixture on LF's HLA repertoire has not been studied before, and the article focuses on the differences in HLA profiles of EF and LF, compares them with modern Europeans, accurately depicts the immune characteristics of the ancients, and demonstrates correlation with the admixture of WHG and the virus selection pressure. The added value to this paper is the newly generated genome-wide data of 83 individuals from six archaeological sites covering the 2300-year transition from the Early (LBK) to the Late Neolithic, which provides a high-quality data reference for the study of ancient DNA in the Neolithic age. However, several concerns are to be considered and addressed.

Major:

**1.**This study has interesting observation on the increased HLA diversity from early farmer to late farmers by generating and analyzing new HLA variation data of 79 individuals. The authors correlate such diversity increase to WHG admixture, though the correlation between HLA diversity and WHG admixture does not necessarily mean causal. It would be nice to see more solid evidence in the correlation or causal test.

We have now performed local ancestry inference analysis in imputed and well covered LF individuals, for which an excess of WHG ancestry was observed (please see our reply #**9.2** to Reviewer #1 for more details; **Figure 4**).

**2.**The authors also reported that both EF and LF exhibited a relatively narrow HLA allele spectrum compared to today, which in authors' opinion coincides with sparse traces of

pathogen DNA, potentially indicating a lower pathogen pressure at the time. It is worth discussing whether it is the fact of sparse traces of pathogen DNA or just not big enough dataset or sampling bias (in the later two cases we can rarely find pathogen DNA)..

We are aware of the inherent problems of pathogen screening in ancient populations (e.g., only bloodborne pathogens can be detected). However, we and colleagues have screened many Neolithic samples (>4000), but have only identified a few sporadic cases of infections (e.g., with HBV, parvovirus B19, *Y. pestis, Salmonella, H. pylori*) across Eurasia. For instance, Sikora et. al., 2023 have reported a low number of pathogens in the Neolithic, which only increased in the Bronze Age. We have also observed a large pathogen load later in the early Middle Ages (Bonczarowska et. al., 2022). Therefore, we are confident that the lack of pathogens in the Neolithic is not due to a technical issue or sampling bias.

We have clarified this aspect in the Discussion in line 328 and lines 329-330. We have also added that the low pathogen loads in the Neolithic is consistent with findings from ancient populations showing that genetic adaptation of humans to infectious diseases only occurred after the beginning of the Bronze Age.

**3.1**The authors also found drastic changes in Y-haplogroups from EF to LF and sex-biased admixture in LF. Regarding the sex bias, has the authors tested with other approaches, apart from Rx/A ratio.

We initially used the Q statistic, which is based on Fst values from autosomes and the X chromosome, to investigate signals of sex-biased admixture as described in the Results in lines 166-170. Apart from the Y-haplogroup changes, Q statistic and the RX/A ratio, we did not test other approaches.

**3.2** I wonder how would the data quality (e.g. X-chromosome coverage) affects the robustness of estimates?

We have now investigated the influence of X-chromosome coverage on the accuracy of our ancestry estimates. To do this, we selected a set of 15 individuals with relatively high X-chromosome coverage (i.e., >4,000 SNPs). We then systematically down sampled the X-chromosome data to 1,000 SNPs, repeating this process 1,000 times. Using supervised ADMIXTURE, we generated an empirical distribution of ancestry estimates from the down sampled data. Finally, we compared these estimates to those derived from the full set of SNPs, calculating the r2 value to assess the consistency and robustness of the ancestry estimates (**Figure 6**).

We would like to note that while we have whole-genome shotgun sequencing data for our samples, the published reference individuals used for admixture modelling predominantly have only capture data available. As a result, our analysis on the X-chromosome is limited to SNPs from the 1240K panel.

**Figure 6.** Accuracy of ancestry estimation: distribution of r2 for ancestry estimates on the X chromosome from 1000 down samples.

**4.1**In the analysis of ROH fragments and effective population size, EF has a shorter total length of ROH and a larger effective population size than LF, and according to the change of haplotypes ratio on the sex chromosome, so the author believed that there was consanguineous marriage in LBK (that is, the period from EF to LF). Counting the difference between EF and LF, the author proposed a hypothesis that EF itself had the custom of consanguineous marriage, which led to the decrease of effective population and the accumulation of Roh fragment length until the LF formed as a result of mixing with WHG , which failed to improve this situation significantly. Given this problem, I think there are two aspects to be analyzed: First, we can view the existing EF data to be subdivided in time scale, and check whether the late EF population has a smaller effective population size or a longer Roh segment than the earlier EF population (regardless of environmental changes), which, to some extent, can support the hypothesis of consanguineous marriage in LBK period;

We would like to clarify that we do not claim consanguineous marriage in EF. Rather, in line 253-256 we stated the following:

“The genetic continuity throughout the LBK indicates long-lasting intracultural mating practices. However, close-kin mating was likely prevented as the analysis of ROH and Ne suggested a large group size and a wide partner exchange network as reported previously.”

By “intracultural mating practices” we mean mating within the larger LBK community. Furthermore, please see Sup. Fig 12C for changes in ROH over time. Since there are no apparent changes in the Early Neolithic in that region, consanguineous practices are unlikely.

**4.2** Second, we can check the Y chromosome typing status of WHG to answer the question why with a large number of WHG mixed in, especially when the author also made sex-bias analysis at the same time and believed that the large number of WHG mixed in favor of men, there is still only one y haplotypes?

Thank you for pointing out an important aspect that we would otherwise have missed. In response to your comment, we took a closer look at the Y-chromosome haplogroups and discovered interesting patterns. We have now included a new paragraph in the Results and Discussion addressing these points (lines 154-161, 273-281).

While we initially reported only the Y macro-lineage "I" in Sup. Figure 9**,** the Sup. Data 1 provides higher-resolution haplotypes for 16 men. These 16 individuals carry just three lineages: I2a1a (n=5), I2a2 (n=1), and I2c (n=10), all of which are associated with WHG populations (Posth et. al., 2023; Szécsényi-Nagy et. al., 2015). Notably, there are dominant lineages within the WBC-associated Late Neolithic communities that vary between sites—for instance, I2c is prevalent in Altendorf and Niedertiefenbach, whereas I2a1a dominates in Warburg.

We have not quantified the number of male WHG ancestors. Therefore, we cannot state the number was large. However, the long-term effects of this admixture were still noticeable generations later in WBC (~36% WHG ancestry).

**5.**The author believes that the frequency change of LF compared with EF in the HLA region is mainly from the mixing of WHG, and the basis is that, in line 253, DQB1 locus(within HLA-II) is enriched in the individuals with WHG ancestors in the Neolithic age, while the major frequency 252 changes (>20%) between EF and LF mainly affect HLA class II alleles. Through the previous analysis, we can see that there are some WHG individuals in this study (loschbour Luxembourg, Bichon Switzerland, and one individual from Mont aim é/paris102 basin), I think these individuals can be directly used together with EF to calculate the introgression fragments of the two in LF, which can more directly explain the causal relationship.

Reliable HLA genotyping requires a targeted enrichment as we have done in this study. Unfortunately, it is not possible to perform such enrichment for the WHG individuals that are mentioned here. However, we were able to provide indirect support for the introgression of specific HLA alleles based on our local ancestry analysis. For details see our reply **#9.2** to Reviewer #1; **Figures 4-5**.

**6.**The typing of HLA uses a combination of OptiType and TARGT pipeline. Although TARGT is a method for ancient and modern shotgun sequence data with moderate to low sequence coverage, OptiType is not. Why is it combined with OptiType, or why is it not combined with more HLA typing methods?

While it is true that OptiType was not designed for low-coverage data, a recent benchmarking study revealed that its performance with low-coverage data is better than other automated algorithms (Thuesen et. al., 2022). Therefore, we have used it in combination with TARGT. Please see our answer **#1** to Reviewer #1 where we expanded on the reliability of the methods used.

**7.**The preservation condition of ancient DNA may have potential impacts on the results. Sensitivity analysis can be performed on samples with different preservation conditions to assess their impact on HLA gene typing and diversity estimation.

The influence of ancient DNA damage as well as coverage on genotyping accuracy has been analyzed in the original manuscript presenting the TARGT pipeline (Pierini et. al., 2020). Manual inspection of aligned reads minimizes genotyping errors associated with DNA damage (please also see our answer **#1** to Reviewer #1).

**8.1**Only alleles consistently called by both methods were included to ensure a higher reliability of the results. However, OptiType can only type class I genes(HLA-A, -B and -C). How are the results for class II (HLA-DPB1, -DQB1 and -DRB1) handled?

For HLA class II, only TARGT was used.

**8.2** Are there any statistics on the consistency of typing between OptiType and TARGT? If it's not very high, it may indicate that even the loci with consistent typing are not very reliable. It is suggested to check the read coverage and depth at several loci for enhanced reliability.

Yes. Consistency between OptiType and TARGT is around 93% based on Pierini et. al., 2020, which is fairly high. We further increase the reliability of TARGT by having two independent calls from two researchers. In addition, please see our reply **#1** to Reviewer #1.

We have now expanded the Methods with more information on the reliability of both HLA calling methods used (lines 552-561).

**9.**HLA polymorphism increases significantly with sample size. For the alleles with significant frequency differences found in EF, LF, and GER, how to exclude the influence of the number of samples and sampling bias? Multiple random downsamplings could be conducted to determine if the results remain consistent.

As suggested, we generated 100 bootstrap samples (with replacement), each of size n, where n corresponds to the size of the smallest group, which was consistently the EF group (**Figure 7** below). The frequency distributions from these resamples were then compared pairwise between the groups using a two-sided Mann-Whitney U test. The p-values were corrected for multiple testing using the two-stage Benjamini and Hochberg step-up FDR-controlling procedure (TSBH-FDR). All previously identified alleles that showed a significant difference with the Fisher’s exact test (without bootstrapping) remained significant after applying the bootstrapping approach described above (p-values in **Table 1**).

**Figure 7.** Frequency distribution of significantly different alleles across 100 resamples for early farmers (EF), late farmers (LF) and modern Germans (GER).

**Table 1.** HLA alleles exhibiting statistically significant and substantial shifts (p ≤ 0.05, absolute frequency difference ≥ 10%) between early farmers (EF) and late farmers (LF) or between either Neolithic group and modern Germans (GER). The p-values were calculated with Fisher’s exact test and corrected for multiple testing using the two-stage Benjamini and Hochberg step-up FDR-controlling procedure (TSBH-FDR). In blue are the values obtained with the two-sided Mann-Whitney U test after 100 resamples with replacement using size n, where n represents the sample size for the population with smallest size for each locus, which was always the EF group.

**10.**It is advisable to consider and discuss other factors that may influence HLA diversity, such as environmental pressure, in addition to admixture.

We have mentioned pathogen-driven selection in the discussion section in lines 324-330.

minor:

**11.**To solve the problem that the mixed model of WHG estimated the mixing time with dates is not accurate, MultiwaverX software can be used, which supports the construction of a gender-biased mixed model and the construction of a mixed model with continuous mixed gene flow.

Thank you for the suggestion. However, the implementation of MultiwaverX is currently beyond the scope of this study.

**12.**As for the characterization of the diversity of different groups in the HLA region, we can also try to use nucleotide diversity and haplotype diversity.

We opted to use only the Shannon Index measure as it was previously shown that diversity measures based on molecular (i.e. nucleotide diversity) and allelic (i.e. HLA allele names) approaches are highly correlated (Buhler & Mazas, 2011).

**REFERENCES**

Allentoft ME, Sikora M, Refoyo-Martínez A, Irving-Pease EK, Fischer A, Barrie W, et al. Population genomics of post-glacial western Eurasia. Nature. 2024 Jan 11;625(7994):301–11.

Barrie W, Yang Y, Irving-Pease EK, Attfield KE, Scorrano G, Jensen LT, et al. Elevated genetic risk for multiple sclerosis emerged in steppe pastoralist populations. Nature. 2024 Jan 11;625(7994):321–8.

Bonczarowska JH, Susat J, Mühlemann B, Jasch-Boley I, Brather S, Höke B, et al. Pathogen genomics study of an early medieval community in Germany reveals extensive co-infections. Genome Biol. 2022 Dec 13;23(1):250.

Buhler S, Sanchez-Mazas A. HLA DNA Sequence Variation among Human Populations: Molecular Signatures of Demographic and Selective Events. Mokrousov I, editor. PLoS ONE. 2011 Feb 1;6(2):e14643.

Davy T, Ju D, Mathieson I, Skoglund P. Hunter-gatherer admixture facilitated natural selection in Neolithic European farmers. Current Biology. 2023 Apr;33(7):1365-1371.e3.

Goldberg A, Günther T, Rosenberg NA, Jakobsson M. Ancient X chromosomes reveal contrasting sex bias in Neolithic and Bronze Age Eurasian migrations. Proc Natl Acad Sci USA. 2017 Mar 7;114(10):2657–62.

Irving-Pease EK, Refoyo-Martínez A, Barrie W, Ingason A, Pearson A, Fischer A, et al. The selection landscape and genetic legacy of ancient Eurasians. Nature. 2024 Jan 11;625(7994):312–20.

Pierini F, Nutsua M, Böhme L, Özer O, Bonczarowska J, Susat J, et al. Targeted analysis of polymorphic loci from low-coverage shotgun sequence data allows accurate genotyping of HLA genes in historical human populations. Sci Rep. 2020 Apr 30;10(1):7339.

Posth C, Yu H, Ghalichi A, Rougier H, Crevecoeur I, Huang Y, et al. Palaeogenomics of Upper Palaeolithic to Neolithic European hunter-gatherers. Nature. 2023 Mar 2;615(7950):117–26.

Ringbauer H, Novembre J, Steinrücken M. Parental relatedness through time revealed by runs of homozygosity in ancient DNA. Nat Commun. 2021 Sep 14;12(1):5425.

Sikora M, Canteri E, Fernandez-Guerra A, Oskolkov N, Ågren R, Hansson L, et al. The landscape of ancient human pathogens in Eurasia from the Stone Age to historical times [Internet]. 2023 [cited 2024 Sep 4]. Available from: http://biorxiv.org/lookup/doi/10.1101/2023.10.06.561165

Szécsényi-Nagy A, Brandt G, Haak W, Keerl V, Jakucs J, Möller-Rieker S, et al. Tracing the genetic origin of Europe’s first farmers reveals insights into their social organization. Proc R Soc B. 2015 Apr 22;282(1805):20150339.

Thuesen NH, Klausen MS, Gopalakrishnan S, Trolle T, Renaud G. Benchmarking freely available HLA typing algorithms across varying genes, coverages and typing resolutions. Front Immunol. 2022 Nov 8;13:987655.

**2^nd^ round**

**Reviewer 1**

The authors have effectively addressed the majority of the points raised in the previous round of revision. I especially appreciated the inclusion of the new Local Ancestry Inference (LAI) analysis, which strengthens the manuscript’s claims. This analysis provides compelling evidence for the feasibility of applying such approaches using high-coverage ancient genomes from Europe as sources, a growing resource in recent years.

However, there are still several major points that need to be addressed, which currently prevent the manuscript from being suitable for publication.

My main concerns:

-The computational workflows and scripts used to generate the main figures must be openly shared to ensure transparency and replicability. I appreciate the addition on the materials and method underlined in the answer #2, which are fundamental to understanding the procedure conducted in this study. However, I strongly recommend making this information available, in a more detailed way, in a public repository (e.g., GitHub or another suitable platform) and not leaving it inaccessible. The phrase "available upon request" contradicts the principles of open science.


-The discussion section needs revision, it should be more focused and go beyond simply summarising the results. A more precise and critical discussion, taking the insights and comparisons with previous studies focused on the same genomic region would strengthen the manuscript. On this perspective I recommend to add a few lines about the link between DBQ1 and high celiac disease risk which was recently linked to the introduction of agriculture in Europe (https://www.biorxiv.org/content/10.1101/2024.09.14.613021v1.full). These gene is widely discussed in the current discussion of the manuscript.


-Overall, the figures in the manuscript lack details and sufficient explanation in the captions, and some include redundant or unnecessary information. I will provide a few examples, starting with the response the authors gave to a concern I raised during the previous round of revisions.
--In response #27, the authors refer to Figure 3, which they describe as the previous supplementary figure 14. However, Figure 3 is now the Local Ancestry (LAI) analysis, which was not part of the previous version. They also refer to Figures 4 and 5 as showing the results of the LAI analysis, but this is incorrect. Figure 4 represents the Shannon index, while Figure 5 does not exist in the manuscript. I suggest reorganising the figures: include the two panels of LAI results as a new Figure 3, as they intended in the revision. This figure should have the revised manuscript Figure 3 and the comparison of the mean WHG ancestry across the MHC region as coloured boxplot which was only shown in the answer to reviewers.

--Furthermore, I recommend improving the quality of Figure 4, perhaps by using colour to enhance clarity. Regarding this analysis (and related to response #8), the authors should clarify what they mean by "resampling with replacement." (i.e., If the sample size is 14 for HLA-DRB1 in EF, what exactly are they resampling, especially if they are using the max number of individuals in that group?)

--QpAdm results should be incorporated into Figure 1 as a new panel to confirm the population structure, or they could be included in a separate figure highlighting the Niedertiefenbach samples in relation to the newly sequenced samples (i.e. the EFs from new archeological sites). This is an important part of the manuscript and should be visually represented.

--In Figure 2, the authors need to specify what is plotted on the Y-axis. Is it the frequency of alleles in specific HLA gene groups? I was able to deduce this from supplementary figure X, but the authors must explicitly state what these frequencies represent. This is especially important as the frequencies are discussed extensively throughout the manuscript, and statistical tests are applied in both the results and methods sections.

--Supplementary Fig. 15 has a wrong legend, DQB1 04:02 is plotted but in the caption authors refer to 04:01

-In general, the authors should revise the quality and clarity of their figures and supplementary Figures. At present, only Figure 1 seems ready for publication.


Minor points:

-In response #1, please clarify whether you are referring to transitions or transversions. Both terms were used in the response, which needs clarification.

-In response #8.1, please clarify why only TARGT was used for three key genes—HLA-DPB1, HLA-DQB1, and HLA-DRB1—given their importance in the risk of several pathologies.

-Line 386, "ALN" should be corrected to "aln"

-Regarding response #18, contrary to what the authors state, it appears that the Niedertiefenbach samples were used in the population structure analysis, as indicated by both the figures and the table (Table 1). Since a few samples were generated in this study, I believe the archaeological site should not be introduced in only one section without briefly mentioning it when analysing/introducing these samples for the first time in the manuscript (i.e, during the population structure). To avoid confusion, all data (newly published samples and those from Immel 2017) should be analysed together from the beginning. If the authors choose not to do this, they should clearly specify why. Then, a separate section can be dedicated to the Niedertiefenbach site, where they introduce and discuss the evidence specific to this site/region. Finally, I had previously suggested including this information in a new figure of the main text maybe exploiting qpAdm results.

**Authors’ response**

We thank again Reviewer #1 for his/her comments. Below, we address each point individually. Reviewer #1's comments are prefixed with “-”, and our responses are indicated by “R:”. Changes made to the manuscript are highlighted in red.

___________

Major points:

- The computational workflows and scripts used to generate the main figures must be openly shared to ensure transparency and replicability. I appreciate the addition on the materials and method underlined in the answer #2, which are fundamental to understanding the procedure conducted in this study. However, I strongly recommend making this information available, in a more detailed way, in a public repository (e.g., GitHub or another suitable platform) and not leaving it inaccessible. The phrase "available upon request" contradicts the principles of open science.

R: The code to generate the requested figures is deposited in GitHub

(https://github.com/dasilvan9/HLA_diversity_in_the_Neolithic) and linked to Zenodo

(https://doi.org/10.5281/zenodo.14185098).

-The discussion section needs revision, it should be more focused and go beyond simply summarising the results. A more precise and critical discussion, taking the insights and comparisons with previous studies focused on the same genomic region would strengthen the manuscript. On this perspective I recommend to add a few lines about the link between DBQ1 and high celiac disease risk which was recently linked to the introduction of agriculture in Europe (https://www.biorxiv.org/content/10.1101/2024.09.14.613021v1.full). These gene is widely discussed in the current discussion of the manuscript.

R: We have extended the discussion to add insights into the history of specific HLA alleles (see below). In addition, we have shortened and rephrased parts of the discussion to be more focused. Following the reviewer’s advice, we have expanded on the origin and spread of the DQB1*02:01 allele which is associated with celiac disease. We would also like to point out that to the best of our knowledge we have now cited all the paleo-genetic studies that analyzed HLA data. Importantly, only a few of those studies were able to resolve specific alleles, while the majority could only provide results on the gene level. However, we focused on higher-resolution analysis at the allele level, targeting specific alleles that exhibited significant temporal changes and are relevant to disease susceptibility in modern populations. We have discussed these alleles in more detail (B*27:05, B*51:01, DRB1*08:01, DRB1*13:01, DRB1*15:01, DQB1*02:01, DQB1*04:02, DQB1*06:02, DQB1*06:03).

-Overall, the figures in the manuscript lack details and sufficient explanation in the captions, and some include redundant or unnecessary information. I will provide a few examples, starting with the response the authors gave to a concern I raised during the previous round of revisions.

--In response #27, the authors refer to Figure 3, which they describe as the previous supplementary figure 14. However, Figure 3 is now the Local Ancestry (LAI) analysis, which was not part of the previous version. They also refer to Figures 4 and 5 as showing the results of the LAI analysis, but this is incorrect. Figure 4 represents the Shannon index, while Figure 5 does not exist in the manuscript. I suggest reorganising the figures: include the two panels of LAI results as a new Figure 3, as they intended in the revision. This figure should have the revised manuscript Figure 3 and the comparison of the mean WHG ancestry across the MHC region as coloured boxplot which was only shown in the answer to reviewers.

R: The labeling of the figures has been corrected. As recommended, Figure 3 contains now two panels showing the LAI results. Figure 3B (boxplot) is now colored.

--Furthermore, I recommend improving the quality of Figure 4, perhaps by using colour to enhance clarity. Regarding this analysis (and related to response #8), the authors should clarify what they mean by "resampling with replacement." (i.e., If the sample size is 14 for HLA-DRB1 in EF, what exactly are they resampling, especially if they are using the max number of individuals in that group?)

R: Figure 4 was remade using different colors for each group.

The method of resampling with replacement allows for creating multiple bootstrap samples of the desired size by randomly selecting individuals that are eligible to be chosen more than once. Therefore, in the example of HLA-DRB1, resampling with replacement for EF means that each individual in the original sample of 14 could appear more than once in a resampled dataset. We added this explanation to the Methods.

--QpAdm results should be incorporated into Figure 1 as a new panel to confirm the population structure, or they could be included in a separate figure highlighting the Niedertiefenbach samples in relation to the newly sequenced samples (i.e. the EFs from new archeological sites). This is an important part of the manuscript and should be visually represented.

R: The qpAdm results were incorporated as panel D of Figure 1. The Niedertiefenbach samples are included in all panels of Figure 1.

--In Figure 2, the authors need to specify what is plotted on the Y-axis. Is it the frequency of alleles in specific HLA gene groups? I was able to deduce this from supplementary figure X, but the authors must explicitly state what these frequencies represent. This is especially important as the frequencies are discussed extensively throughout the manuscript, and statistical tests are applied in both the results and methods sections.

R: The Y axis represents allele frequencies calculated for each locus (HLA-A, -B, -C, - DQB1, -DRB1 and -DPB1) separately. We edited the Figure 2 legend accordingly.

--Supplementary Fig. 15 has a wrong legend, DQB1 04:02 is plotted but in the caption authors refer to 04:01

R: Supplementary Figure 15 is now the main Figure 3B. The legend was corrected to refer to DQB1*04:02.

-In general, the authors should revise the quality and clarity of their figures and supplementary Figures. At present, only Figure 1 seems ready for publication.

R: All main figures have been updated for enhanced visual quality, and captions have been revised for improved clarity. Colors for EF, LF, and GER have been standardized across Figures 1, 2, and 4 to maintain consistency. We have also made the following improvements to the supplementary figures:

Fig S2: Added results for Niedetiefenbach and set a fixed range on the Y-axis for easier visualization and comparison.

Fig S3: Rephrased caption to improve clarity.

Fig S4: Updated caption to provide a more detailed explanation of the figure.

Fig S5: Replaced previous panels with a consolidated plot displaying admixture estimates from various models as boxplots.

Fig S6: Rephrased caption to improve readability.

Fig S8: Applied distinct color palettes for mtDNA and Y chromosome haplogroups for more precise differentiation and updated the caption.

Fig S9: Enhanced plot quality by increasing its width and removing redundant elements from the plot and caption.

Fig S10: Improved overall visual quality of the figure.

Fig S11: Added more detail to caption.

Fig S12: Enhanced quality and increased the visibility of boxplots within the violin plots. Additionally, we have included two new figures (Figs S14 and S15) that were only shown in the rebuttal letter in response to Reviewer 2’s questions #3.2 and #9, along with corresponding updates in the Results and Methods sections. We also added a new column to Table S4 and updated its title to reflect the addition of Fig S14 to the manuscript.

___________

Minor points:

-In response #1, please clarify whether you are referring to transitions or transversions.

Both terms were used in the response, which needs clarification.

R: For clarity, we have reworded the section of response #1 to only use the term “transversions”:

“Although using only transversions is a common practice in SNP-based aDNA analysis, applying it to our approach would be counterproductive. Only using transversions would make it impossible to distinguish some HLA alleles, especially at resolutions higher than 1-field. Also, many polymorphic positions across the HLA genes do not fit the classical biallelic categorization, as more than two nucleotides can be observed in one position. Therefore, only using transversions would result in a bias toward calling alleles that are differentiated mainly by transversions.”

-In response #8.1, please clarify why only TARGT was used for three key genes—HLA-DPB1, HLA-DQB1, and HLA-DRB1—given their importance in the risk of several pathologies.

R: For HLA class II genes, there are no suitable open-source tools for typing low coverage ancient genomes. Therefore, we could only use TARGT. This was already explained in the Methods section in the previously submitted version.

-Line 386, "ALN" should be corrected to "aln"

R: This correction was applied to the Methods.

-Regarding response #18, contrary to what the authors state, it appears that the Niedertiefenbach samples were used in the population structure analysis, as indicated by both the figures and the table (Table 1). Since a few samples were generated in this study, I believe the archaeological site should not be introduced in only one section without briefly mentioning it when analysing/introducing these samples for the first time in the manuscript (i.e, during the population structure). To avoid confusion, all data (newly published samples and those from Immel 2017) should be analysed together from the beginning. If the authors choose not to do this, they should clearly specify why. Then, a separate section can be dedicated to the Niedertiefenbach site, where they introduce and discuss the evidence specific to this site/region. Finally, I had previously suggested including this information in a new figure of the main text maybe exploiting qpAdm results.

R: We followed the Reviewer’s recommendation and included the Niedertiefenbach samples from the beginning (previously published and newly generated ones). Corresponding updates have been made to all sections to reflect this change. Additionally, as requested above, we included qpAdm with Niedertiefenbach as a new panel in Figure 1.
